# GATA3-driven CDC20 activation facilitates tumor growth and metastasis through suppressing Bax/Bcl-2 ratio and boosting EMT process in neuroblastoma

**DOI:** 10.1016/j.jbc.2026.113550

**Published:** 2026-09-12

**Authors:** Xiaona Fang, Yingxia Lan, Mengzhen Li, Weiji Xie, Lei Mao, Jinlin Huang, Qiuting Li, Yu Zhang, Mengjia Song, Yi Que, Suying Lu, Juan Wang, Junting Huang, Jia Zhu, Yi Wang, Feifei Sun, Yizhuo Zhang

**Affiliations:** 1 Department of Pediatric Oncology, Sun Yat-sen University Cancer Center, Guangzhou, China; 2 State Key Laboratory of Oncology in South China, Guangdong Provincial Clinical Research Center for Cancer, Collaborative Innovation Center for Cancer Medicine, Sun Yat-sen University Cancer Center, Guangzhou, China; 3 Department of Anesthesiology, Sun Yat-sen University Cancer Center, Guangzhou, China; 4 Department of Pathology, Sun Yat-sen University Cancer Center, Guangzhou, China

**Keywords:** cell division cycle 20, GATA-binding protein 3, MYCN Proto-oncogene, tumorigenesis, metastasis, neuroblastoma

## Abstract

Neuroblastoma (NB) is a heterogeneous pediatric malignancy in which many high-risk tumors lack *MYCN* amplification, necessitating the identification of alternative molecular drivers. Here, integrative transcriptomic analyses of *MYCN*-nonamplified NB cohorts identified cell division cycle 20 (CDC20) as a candidate associated with high-risk disease and metastasis. *CDC20* expression was elevated in aggressive NB across subtypes and correlated with adverse clinical outcomes, with particularly informative prognostic value in *MYCN*-nonamplified patients. Functional studies established *CDC20* as a potent oncogene that promotes proliferation, migration, apoptosis resistance, and tumor progression in NB models, regardless of *MYCN* status. Pharmacological inhibition of CDC20 further supported CDC20 as a potential therapeutic vulnerability in NB. Mechanistically, we found that the transcription factor GATA3 directly bound a specific motif within the *CDC20* promoter, as validated by ChIP-qPCR and site-directed point mutagenesis, and transcriptionally activated its expression. Reciprocal genetic epistasis experiments further showed that CDC20 depletion attenuated GATA3-driven oncogenic phenotypes, whereas restoring CDC20 rescued the impaired growth, migration, and survival caused by GATA3 depletion. At the downstream level, CDC20 facilitates NB progression *via* dual mechanisms: suppressing the pro-apoptotic Bax/Bcl-2 ratio and inducing an epithelial-mesenchymal transition-like phenotype conducive to metastasis. Collectively, our study delineates a previously uncharacterized GATA3-CDC20 regulatory axis that promotes NB progression and identifies CDC20 as a potential therapeutic vulnerability across NB subtypes, while highlighting its particular potential for risk stratification in *MYCN*-nonamplified disease.

Neuroblastoma (NB) is the most common extracranial malignant solid tumor in children, accounting for approximately 10% to 15% of pediatric cancers and 15% of childhood cancer-related deaths (1). High-risk NB, as defined by the Children's Oncology Group (COG) risk stratification system, includes patients with *MYCN*-amplified tumors, those ≥ 18 months of age with International Neuroblastoma Staging System (INSS) stage 4 disease, or other unfavorable clinical/biological features. The 5-year survival rate for patients with high-risk NB remains less than 50%. High-risk NB is characterized by significant tumor heterogeneity and complex biology, which complicates treatment and leads to poor prognosis (2). Diverse genetic and molecular profiles contribute to the multifaceted tumor heterogeneity and unfavorable outcomes (3). In recent years, with more focus on the underlying molecular mechanisms of high-risk and relapsed NB, more innovative and therapeutic targeted strategies have been studied and exploited. For instance, the identification of gain-of-function *ALK* mutations in familial and sporadic NB has led to the clinical development of ALK inhibitors, such as Lorlatinib, representing a successful targeted therapeutic strategy for a molecularly defined subset of patients (4). Besides, the loss of function of neurofibromin 1 in high-risk NB has been recognized as a potential target for SHP2 inhibition (5). Studies have shown that SHP2 inhibitors can effectively block tumor growth in models with NF1 loss, offering a new avenue for the treatment of relapsed NB cases (6). While considerable progress has been made in deciphering NB's biological mechanisms, obstacles remain (7). Continued research into the molecular underpinnings and the development of novel therapeutic strategies will be beneficial to improve the prognosis of patients with high-risk NB.

*MYCN* amplification is a critical genetic aberration in a subgroup of high-risk NB, strongly associated with rapid tumor growth, aggressive behavior, advanced disease stages, and unfavorable prognosis (8, 9). Approximately 40% of high-risk NB harbor *MYCN* amplification, yet most patients do not exhibit this genetic feature, suggesting that additional genetic or molecular abnormalities drive the pathogenesis and progression of high-risk NB without *MYCN* amplification (10). Recent studies have highlighted the importance of other genetic and molecular factors in determining the prognosis of patients with high-risk NB. For instance, the expression of MYC, a family member of MYCN, has been recognized as a strong oncogenic driver in certain high-risk NB cases (11). This activation occurs through mechanisms such as enhancer hijacking and focal enhancer amplification, and the clinical outcomes for patients with high MYC expression are similar to those with *MYCN* amplification (11). This suggests that MYC could serve as an alternative biomarker for aggressive disease in the absence of *MYCN* amplification. Notably, the identification of other genetic alterations and functional signatures provides valuable insights into the biology of high-risk neuroblastoma and highlights the potential for developing more targeted therapeutic strategies for patients lacking *MYCN* amplification.

While *MYCN* amplification is a critical driver in a subset of high-risk NB, the molecular underpinnings of aggressive disease in *MYCN*-nonamplified NB remain poorly characterized, highlighting the need to identify alternative oncogenic drivers. We therefore performed integrative transcriptomic analyses including Weighted Gene Co-expression Network Analysis (WGCNA) and gene enrichment analysis focused on *MYCN*-nonamplified NB. Genes independently associated with high-risk classification and metastatic disease were integrated to prioritize candidates linked to both aggressive phenotypes, identifying CDC20 as a leading candidate. CDC20, a cell division cycle protein, has been reported as a hallmark of carcinogenesis and cancer progression in several cancers. In osteosarcoma, for instance, CDC20 has been shown to promote cell proliferation and invasion while inhibiting apoptosis, suggesting its potential oncogenic role in various cancers (12). This oncogenic role is partly due to the regulation of proteins such as Bim and p21, which are involved in cell cycle control and apoptosis (13). Besides, the potential of CDC20 to be a prognostic factor and therapeutic target was reported in hematological malignancies as well (14). However, the specific role of CDC20 in NB has not been extensively investigated. We subsequently established the functional relevance of CDC20 using NB models with different *MYCN* amplification statuses and evaluated its therapeutic vulnerability through complementary genetic and pharmacological approaches. Mechanistically, we identified GATA3 as its direct transcriptional activator and elucidated that CDC20 promotes NB progression by inhibiting apoptosis and enhancing cellular plasticity. Pharmacological inhibition of CDC20 attenuated tumor growth and metastasis. Together, our findings establish a previously uncharacterized GATA3-CDC20 regulatory axis that contributes to NB progression and support CDC20 as a potential therapeutic vulnerability across NB subtypes, while highlighting its particular potential for risk stratification in *MYCN*-nonamplified disease.

## Results

### Identification of *CDC20* as a candidate oncogene in *MYCN*-nonamplified high-risk and metastatic NB

To delineate molecular drivers underlying high-risk and metastatic NB without *MYCN* amplification, we performed Weighted Gene Co-expression Network Analysis (WGCNA) using the expression matrix obtained from NB tumors in the GEO database. We defined "high-risk" per COG criteria and "metastatic" as INSS stage 4, which are available in the clinical metadata of the online datasets. Specifically, differentially expressed genes (DEGs) between the high-risk and non-high-risk groups lacking *MYCN* amplification (GSE49710) were determined using the "Limma" R package, applying thresholds of |log2-fold change (FC)| > 0.45 and adjusted *p* value < 0.05. This resulted in a set of 4536 genes for subsequent WGCNA analysis, systematically excluding data from the eight outlier samples by sample clustering (Fig. S1*A*). Gene clustering assigned all transcripts to specific modules, with the gray designation reserved for genes lacking meaningful co-expression relationships (Fig. S1*B*). The optimal soft threshold was determined to be 9, as indicated by the analysis function, achieving a signed R^2^ of 0.85 (Fig. S1*C*). Using WGCNA's dynamic hybrid tree-cutting algorithm and similar module merging, eight discrete modules were identified, and their relationships were visualized using a heatmap (Fig. 1*A*). Among these, the MEyellow module was significantly correlated with high-risk disease in *MYCN*-nonamplified NB (Fig. 1*A*). A scatter plot was generated to visualize the relationship between module membership and gene significance within this module (Fig. 1*B*). Among these genes, 108 key genes associated with high-risk NB were isolated for further analysis (designated Geneset-1) (Fig. 1*B*).

**Figure 1:**
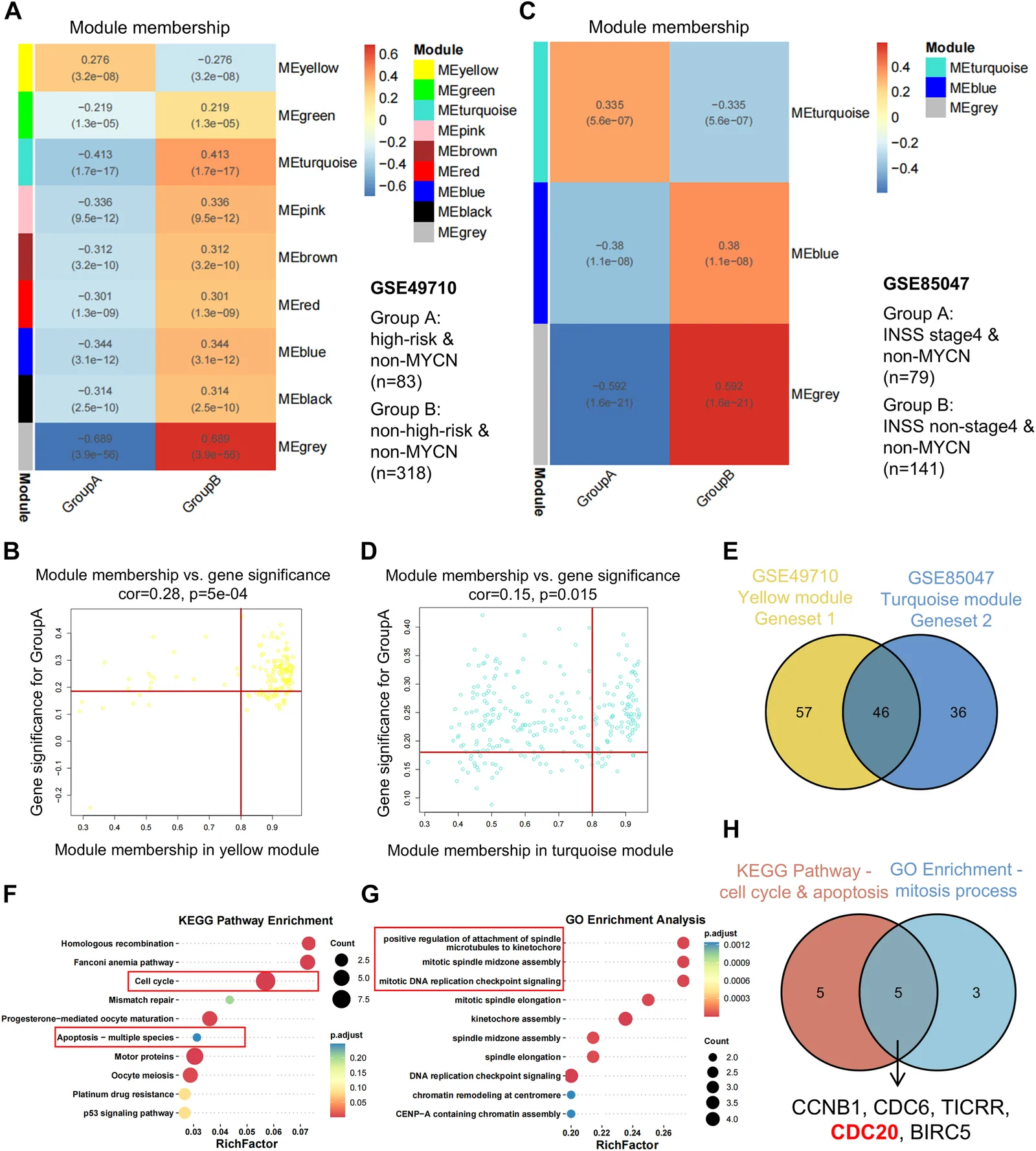
**Identification of *CDC20* as a candidate oncogene in *MYCN*-nonamplified high-risk and metastatic NB.***A*, Heatmap showing the significant correlation between the MEyellow module and high-risk NB diseases independent of *MYCN* amplification. *B*, Relationship between module membership of genes within the MEyellow module and their significance to high-risk NB diseases. *C*, Heatmap indicating the substantial association between the MEturquoise module and NB metastasis phenotype independent of *MYCN* amplification. *D*, Relationship between module membership of genes within the MEturquoise module and their significance to metastatic NB diseases. *E*, Venn plot depicts the overlapping genes from Geneset-1 and Geneset-2. *F*, Enrichment results of the overlapped 46 genes in KEGG terms indicating enrichment in cell cycle and apoptosis pathways. *G*, Enrichment results of the overlapped 46 genes in GO terms highlighting enrichment in the mitosis-associated process, which indicates that these genes may play critical roles in regulating cell proliferation in NB. *H*, Venn plot depicts the five overlapping genes from the enriched genes among these three biological processes. Statistical correlations were evaluated using two-tailed Pearson correlation analysis. Pearson's correlation coefficient (*r*) and the corresponding *p* value are shown in the respective panels. *p* < 0.05 was considered statistically significant.

Similarly, DEGs between the metastatic (INSS stage 4) and the non-metastatic (INSS non-stage 4) groups lacking *MYCN* amplification (GSE85047) were analyzed, and a set of 1638 genes was identified for further WGCNA analysis with an adjusted *p* value<0.05. Within this WGCNA framework, sample clustering identified and excluded the data from the 28 outlier samples (Fig. S1*D*). Hierarchical clustering of all genes produced a gene dendrogram, exhibiting distinct color-coded modules (Fig. S1*E*). Ultimately, by selecting the optimal soft threshold of 22 (Fig. S1*F*), we discerned two distinct modules and their interrelationship was elucidated through heatmap visualization (Fig. 1*C*). Consensus clustering analysis revealed a specific gene module that displayed the highest positive correlation with tumor metastatic potential in patients without *MYCN* amplification, subsequently denoted as Geneset-2 (MEturquoise module, 82 genes) (Fig. 1, *C* and *D*). Similarly, correlation analysis was performed by scatter plotting module membership against gene significance values to evaluate the association between intramodular connectivity and phenotypic relevance (Fig. 1*D*). By overlapping genes from Geneset-1 and Geneset-2, 46 genes were screened out, which may play critical roles in NB cancer aggressiveness independent of *MYCN* status (Fig. 1*E*). These candidates were listed in Table S1. Pathway enrichment analysis based on KEGG terms revealed clusters associated with cell cycle and apoptosis pathways (Fig. 1*F*), while enrichment analysis leveraging Gene Ontology (GO) terms underscored multiple connections to the mitosis process (Fig. 1*G*). Cross-referencing the enriched genes among these three biological processes yielded five genes identified as potential candidates, including *CCNB1*, *CDC6*, *TICRR*, *CDC20*, and *BIRC5* (Fig. 1*H*). Notably, several of these genes have been previously implicated in NB biology. For example, CCNB1, CDC6, BIRC5, and TICRR have been reported to promote NB cell-cycle progression, replication stress, tumor aggressiveness, and to show high expression in *MYCN*-positive NB, respectively, and are all associated with poor clinical outcomes in NB (15, 16, 17, 18). Finally, CDC20 was prioritized for further investigation, as its clinical significance and molecular regulation in NB remain incompletely defined.

### CDC20 is a prognostic biomarker associated with aggressive NB, with distinct significance in *MYCN*-nonamplified patients

To define the clinical significance of CDC20, we analyzed its expression in NB cell lines and tumor tissues from the TARGET and GEO databases. Upregulation of *CDC20* was frequently detected in both *MYCN*-amplified and -nonamplified NB cell lines, with no statistically significant difference between the two groups, compared to normal cell lines in the GSE66586 dataset (Fig. 2*A*). In patient cohorts, *CDC20* expression was significantly higher in high-risk *MYCN*-nonamplified tumors compared to non-high-risk tumors (Fig. 2*B*) and in metastatic (INSS stage 4) *versus* non-metastatic tumors (Figs. 2*C* and S2*A*). Specifically, in patients lacking *MYCN* amplification, *CDC20* was dramatically overexpressed in patients with distant metastases compared to those without (Figs. 2*D* and S2*B*). Also, the expression of *CDC20* was elevated in high-risk tumors *versus* non-high-risk tumors (Fig. S2*C*) and in *MYCN*-amplified tumors *versus* *MYCN*-nonamplified tumors (Fig. S2*D*). These results were reinforced by immunohistochemistry (IHC) staining of CDC20 protein in 98 in-house NB clinical samples (Fig. 2*E*). The clinicopathological correlation of CDC20 expression in NB in the SYSUCC cohort was presented in Table 1. Upregulation of CDC20 was significantly correlated with high-risk diseases (Pearson *χ*2 test, *p* < 0.05) and distant metastases (INSS stage 4) (Pearson *χ*2 test, *p* < 0.001) (Fig. 2*F*). More importantly, Kaplan–Meier analysis revealed that high CDC20 expression predicted worse overall survival (OS), disease-free survival (DFS), and progression-free survival (PFS) in the overall TARGET cohort (Fig. 2*G*), and our in-house independent validation cohort (Fig. 2*H*). Importantly, when the survival analysis was restricted to *MYCN*-nonamplified patients, high CDC20 expression remained a significant predictor of poorer OS and DFS (Fig. 2, *I* and *J*). In contrast, within the *MYCN*-amplified subgroup, high CDC20 correlated only with worse DFS, not OS (Fig. S2, *E* and *F*). Furthermore, within the combined high-risk cohort (both *MYCN*-amplified and -nonamplified), high CDC20 predicted worse DFS but not OS (Fig. S2, *G–H*). CDC20 mRNA and protein levels were consistently elevated in 5/5 NB cell lines compared to normal cord blood mononuclear cells (CBMCs) (Fig. S3, *A–C*). Collectively, these results strongly suggest that high expression of CDC20 is closely associated with NB progression and establishes CDC20 as a potential prognostic biomarker for patients with NB. Specifically, CDC20 can be used to further stratify *MYCN*-nonamplified patients for both high-risk, high metastatic potential and poor prognosis.

**Figure 2:**
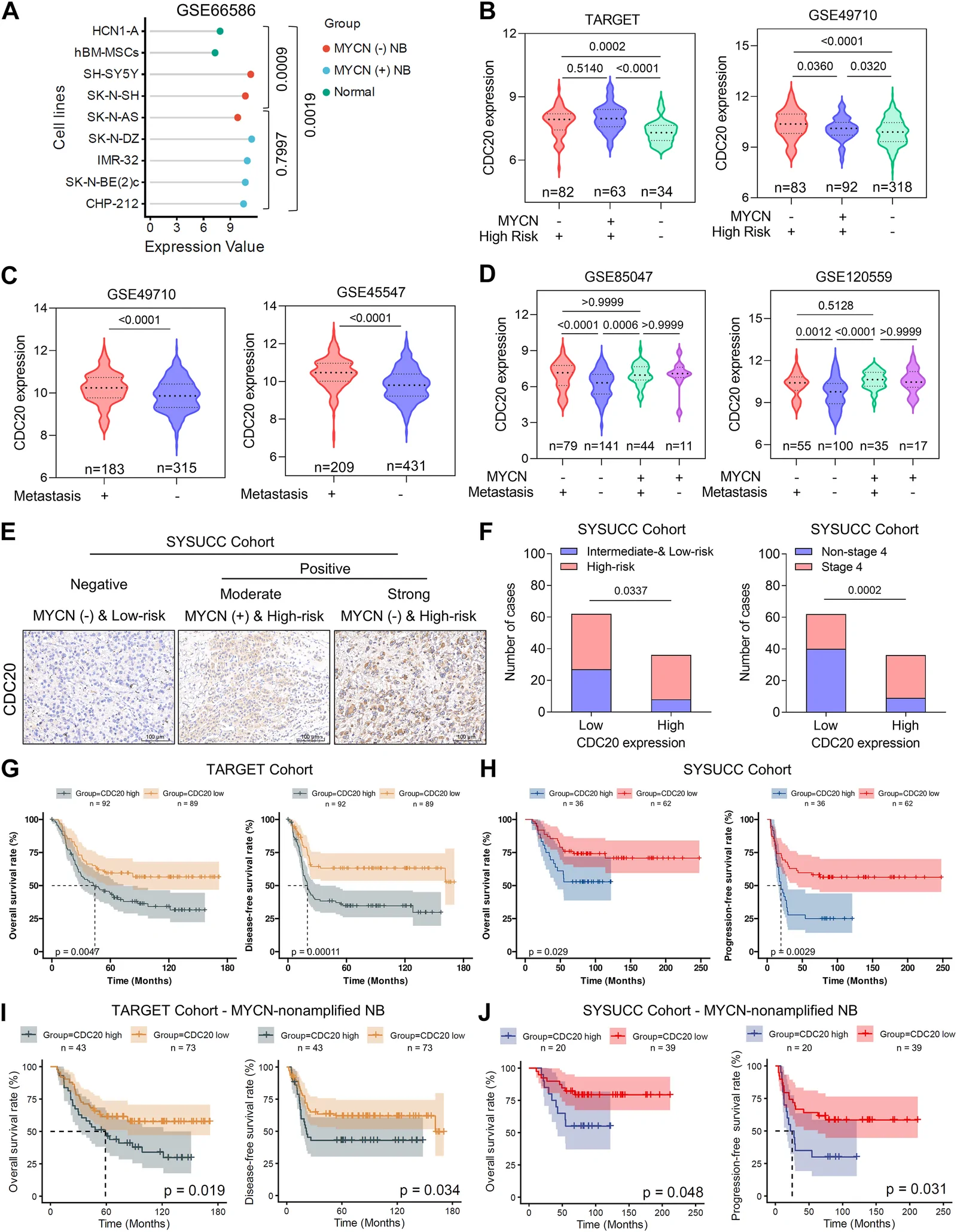
**CDC20 is a prognostic biomarker associated with aggressive NB, with distinct significance in *MYCN*-nonamplified patients.***A*, The expression of *CDC20* was analyzed in the GEO cohort (GSE66586), including normal neuronal cells (HCN1-A) and human bone marrow-derived mesenchymal stem cells (hBM-MSCs), as well as various *MYCN*-amplified and -nonamplified NB cell lines. *B*, *C*, and *D*, The expression of *CDC20* was analyzed in TARGET and GEO cohorts divided according to *MYCN* status and COG risk group (High-risk versus Non-high-risk) (*B*), different clinical INSS stages (*C*), or combined *MYCN* status and clinical INSS stage (*D*). "High-risk" NB, as defined by the Children's Oncology Group (COG) risk stratification system, includes patients with *MYCN*-amplified tumors, those ≥ 18 months of age with International Neuroblastoma Staging System (INSS) stage 4 disease, or other unfavorable clinical/biological features. "Metastasis" disease was defined as INSS stage 4. *E*, Representative images from IHC staining showing the distinct levels of CDC20 protein expression in NB clinical specimens. Scale bars, 100 μm. *F*, High expression of CDC20 was significantly correlated with high-risk diseases and distant metastases (Pearson's chi-square tests, exact *p* values are indicated in the figures). A *p* value < 0.05 was considered statistically significant. The cutoff value for stratifying NB into "low" or "high" CDC20 expression subgroups was calculated according to the overall survival status of patients. *G*–*J*, Kaplan-Meier analysis of the OS, DFS or PFS of patients with NB (*G* and *H*) or MYCN-nonamplified NB (*I* and *J*) from two independent cohorts stratified by the expression level of CDC20. For the TARGET-NB cohort (*G* and *I*), the best-performing threshold was selected as the cutoff value. For the SYSUCC cohort (*H* and *J*), the same cutoff value as in (*F*) was utilized. The *p* values of the log-rank tests were presented. Data were shown as mean ± SD. Statistical analyses were performed using one-way ANOVA with Tukey’s multiple comparisons test for (*A*), Kruskal-Wallis test with Dunn’s multiple comparisons test for (*B*) and (*D*), as well as Mann-Whitney test for (*C*). Exact *p* values are indicated in the figures. A *p* value < 0.05 was considered statistically significant.

**Table 1:** Clinicopathological correlation of CDC20 expression in NB in SYSUCC cohort (n = 98)

Clinical characteristics	Total No. of patients	No. of patients		χ2	*p-*value
		Low (n = 62)	High (n = 36)		
Sex					
Male	62	44	18	4.309	0.038
Female	36	18	18		
Age					
<18 months	18	15	3	3.821	0.051
≥18 months	80	47	33		
INSS stage					
1, 2, 3, 4s	49	40	9	14.23	0.0002
4	49	22	27		
COG risk group					
Low risk	26	22	4	7.27	0.024
Intermediate risk	9	5	4		
High risk	63	35	28		
Chemotherapy before taking pathological specimen					
Yes	62	32	30	9.861	0.002
No	36	30	6		
Primary tumor site					
Adrenal gland	53	39	14	13.22	0.003
Retroperitoneum	32	12	20		
Mediastinum	7	6	1		
Others	6	5	1		
MYCN gene status					
Amplified	19	8	11	5.05	0.08
Nonamplified	59	39	20		
Not available	20	15	5		
Metastases at diagnosis					
Yes	68	40	28	1.886	0.17
No	30	22	8		
Radiotherapy					
Yes	51	26	25	6.906	0.009
No	47	36	11		

### CDC20 potentiates cell proliferation and tumorigenicity in NB

Although the prognostic value of CDC20 was particularly evident in *MYCN*-nonamplified NB, CDC20 expression was not restricted to this subgroup. We therefore used NB cell lines with different *MYCN* amplification statuses in subsequent functional experiments to determine whether the oncogenic effects of CDC20 were dependent on *MYCN* status. To explore the oncogenic role of CDC20 across NB subtypes, various loss-of-function studies were conducted in *MYCN*-amplified cell line SK-N-BE(2) and *MYCN*-nonamplified SK-N-SH and SK-N-AS cells. NB cell lines with *CDC20* knockdown by small-interfering RNA (siRNA) or short-hairpin RNA (shRNA) and CDC20 stable overexpression were established, and the expression of CDC20 was determined by qRT-PCR (Fig. S3, *D* and *E*) and western blotting (Figs. 3, *A* and *B* and S3*F*). CDC20 knockdown significantly impaired cell proliferation (Fig. 3*C*) and colony formation (Fig. 3, *D* and *E*). Conversely, CDC20 overexpression enhanced colony formation (Fig. S3*G*). CDC20-downregulated cells showed enhanced apoptosis ability compared to control cells (Fig. 3, *F* and *G*). To evaluate the *in vivo* pro-tumorigenic function of CDC20, NB cells transfected with shNC or shCDC20 groups were subcutaneously injected into the dorsal flank or right groin areas of NSG mice. Notably, silencing of CDC20 led to a decreased tumorigenesis compared to the control group (Fig. 3, *H*–*K*). Taken together, these data indicate that CDC20 facilitates NB tumorigenicity independently of *MYCN* status both *in vitro* and *in vivo*.

**Figure 3:**
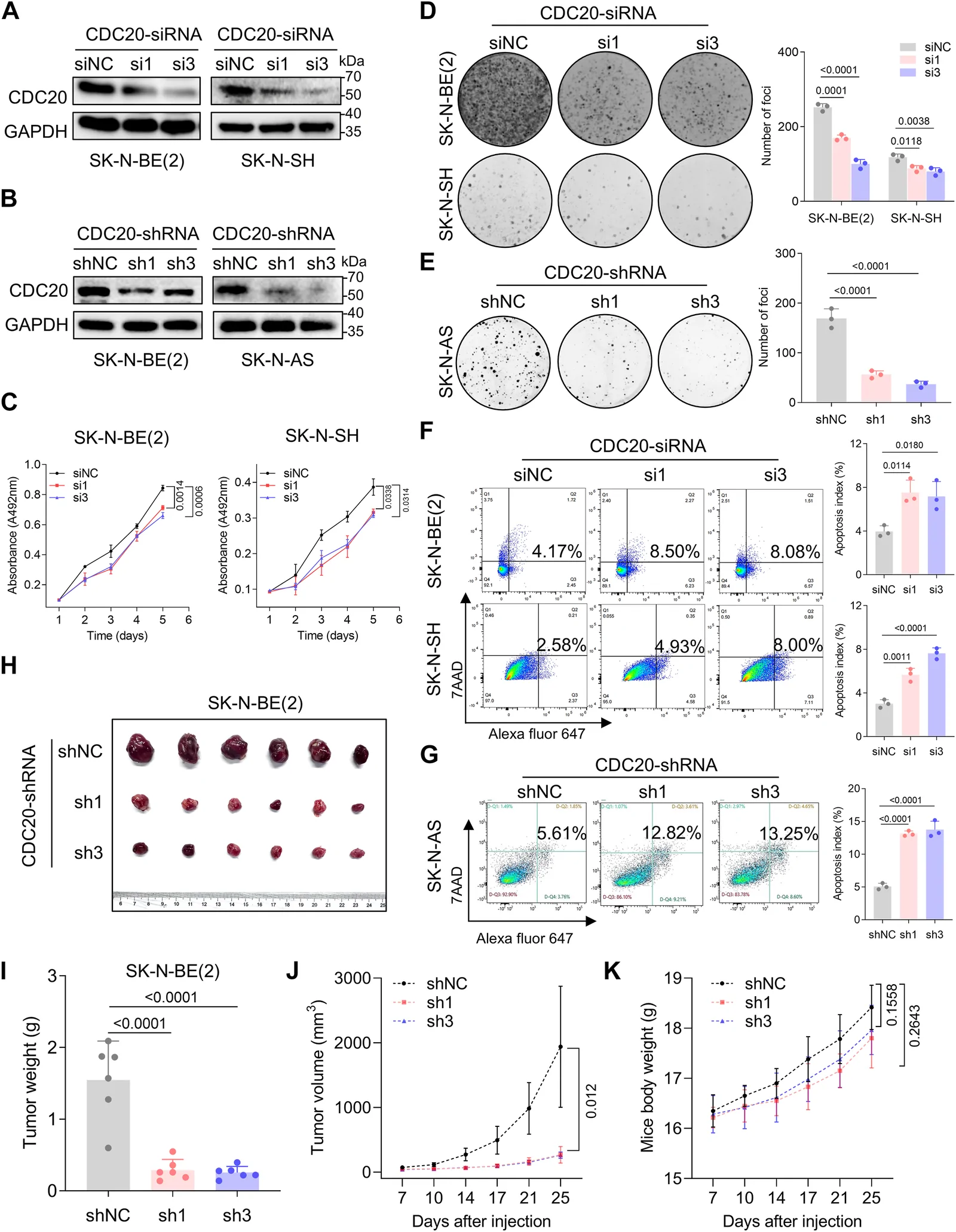
**Knockdown of CDC20 attenuates cell proliferation and tumorigenicity in NB.***A* and *B*, Western blot analysis was used to detect CDC20 expression in CDC20-silenced NB cells. GAPDH was used as a loading control. *C*, The CCK-8 assay was utilized to determine the NB cell proliferation rates. Data were shown as the mean of three independent experiments. *D* and *E*, Representative images of foci formation in monolayer culture. The numbers of foci were calculated and depicted in the bar chart at the *right* panel. *F* and *G*, Representative flow cytometry plots (*left*) and summary (*right*) of apoptotic cells in SK-N-BE(2) and SK-N-SH cells transfected with siNC or siCDC20 and SK-N-AS cells transfected with shNC or shCDC20. *H*, Images of xenograft tumors induced by subcutaneous injection of indicated cells into NSG mice. *I*, Tumor weights were summarized in the bar chart (n = 6). *J*, Tumor growth curves were summarized in the line chart (n = 6). *K*, Mice body weight growth curves were summarized in the line chart (n = 6). For *in vitro* experiments, data are presented as mean ± SD from three independent biological experiments, with individual data points shown. For xenograft experiments, each data point represents 1 mouse, and group sizes are indicated in the corresponding panels or legend. Statistical analyses were performed using two-way ANOVA with Dunnett's multiple comparisons test for (*C*), (*J*), (*K*) and one-way ANOVA with Dunnett's multiple comparisons test for (*D–F*) and (*G–I*). Exact *p* values are indicated in the figures. A *p* value < 0.05 was considered statistically significant.

### CDC20 augments cell migration and tumor metastasis in NB

Given the close association between high CDC20 expression and metastatic status in NB, the effect of CDC20 on NB metastasis was investigated by *in vitro* cell migration assays and *in vivo* mouse experiments using the generated CDC20-downregulated cells. As expected, knockdown of CDC20 significantly suppressed the migratory capabilities of NB cells *in vitro*, as demonstrated by the wound healing assay (Fig. 4*A*) and transwell migration assay (Fig. 4, *B*–*D*). Consistently, overexpression of CDC20 promoted the migratory capacity of NB cells (Fig. S3*H*). Furthermore, the effect of CDC20 on metastasis was investigated by an *in vivo* metastatic mouse model through tail vein injection of different groups of luciferase-labelled NB cells into NSG mice. Knockdown of CDC20 potently diminished tumor metastasis in the liver as shown by bioluminescence imaging (Fig. 4*E*), reduced metastatic nodules on the liver surface (Fig. 4*F*) and histological analysis (Fig. 4*G*). Similar results were observed upon CDC20 knockdown in the *MYCN*-nonamplified SK-N-AS model (Fig. 4*H*). Collectively, these results indicate that CDC20 promotes NB metastasis *in vitro* and *in vivo*.

**Figure 4:**
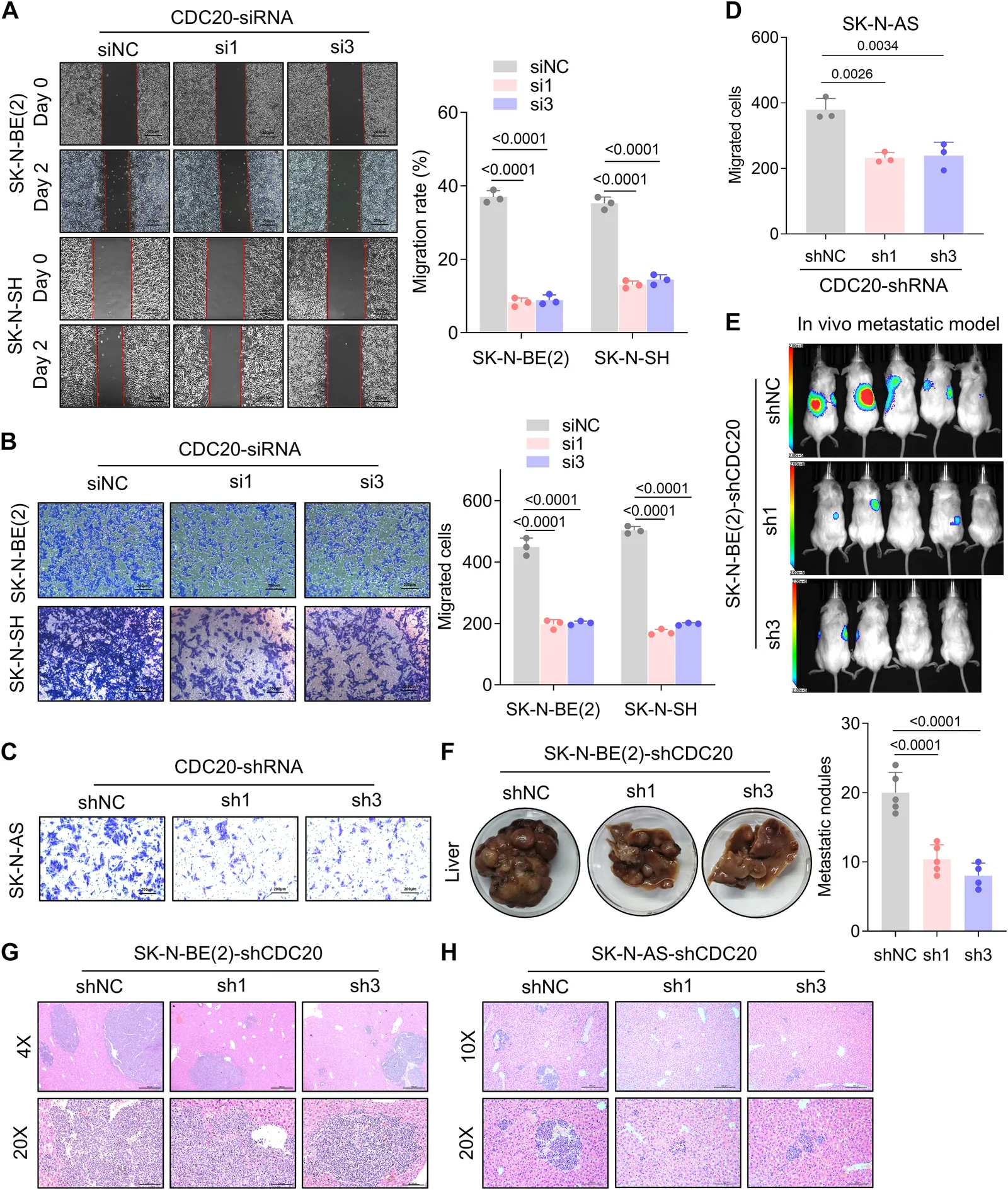
**Knockdown of CDC20 diminishes cell migration and tumor metastasis in NB.***A*, Representative images of wound healing assay in SK-N-BE(2) and SK-N-SH cells transfected with siNC or siCDC20. The migration rate of cells was illustrated in the bar chart. *B*, Transwell migration assay was used to compare cell motility between siNC and siCDC20-transfected SK-N-BE(2) and SK-N-SH cells. Representative images of migration were shown. Scale bars, 200 μm. The number of migrated cells was calculated and depicted in the bar chart. *C*, Representative images of migration from shNC and shCDC20-transfected SK-N-AS cells. Scale bars, 200 μm. *D*, The number of migrated cells was calculated and depicted in the bar chart. *E*, Bioluminescence imaging of NSG mice *via* tail vein injection of luciferase-labeled SK-N-BE(2) cells transfected with shNC or shCDC20 (n = 4–5 mice/group). *F*, *Ex vivo* imaging of livers harvested from the mice is also shown. The metastatic nodules on the surface of the liver were counted. *G*, Representative images of hematoxylin and eosin staining of liver sections mentioned above. Scale bars, 500 μm (*upper panels*) and 100 μm (*lower panels*). *H*, representative images of hematoxylin and eosin staining of liver sections from SK-N-AS cell bearing mice. Scale bars, 500 μm (*upper panels*) and 100 μm (*lower panels*). Data are presented as mean ± SD from three independent biological experiments, with individual data points shown. Statistical analyses were performed using one-way ANOVA with Dunnett's multiple comparisons test for (*A* and *B*), (*D*) and (*F*). Exact *p* values are indicated in the figures. A *p* value < 0.05 was considered statistically significant.

### Pharmacological inhibition of CDC20 reduces tumor growth and metastasis in NB

To further validate the potential therapeutic value of CDC20 in NB, we utilized a pharmacological compound, Apcin (APC inhibitor), which is an inhibitor that targets the CDC20-binding site on the mitotic E3 ligase anaphase-promoting complex/cyclosome (APC/C), thereby blocking the interaction between APC/C and CDC20. Previous studies have revealed that Apcin treatment could suppress cell growth, cell invasion and mobile abilities, as well as induce significant apoptosis in pediatric tumors such as osteosarcoma (13). Data from the Genotype-Tissue Expression (GTEx) database demonstrated that *CDC20* was absent or present at low levels in most normal organs and cell types, supporting its propensity as a selective therapeutic target (Fig. S4*A*). In the present study, Apcin treatment yielded a half-maximal inhibitory concentration (IC50) value of 150∼200 μM in NB cells (Fig. 5*A*). To further evaluate the therapeutic potential of targeting CDC20, we examined the effects of Apcin in NB cells. Consistent with inhibition of APC/C-CDC20 activity, Apcin treatment resulted in dose-dependent accumulation of Cyclin B1 and securin, two established APC/C-CDC20 substrates, in SK-N-BE(2) and SK-N-AS cells (Fig. S4*B*). Then *in vitro* functional assays and *in vivo* subcutaneous mouse model were performed using NB cells after intervening with concentrations of Apcin. CDC20 inhibition by Apcin treatment significantly attenuated cell clonogenicity (Fig. 5*B*) and induced apoptosis (Fig. 5*C*) of NB cells *in vitro* in a dose-dependent manner. Furthermore, the cell migration capabilities were dramatically reduced after Apcin treatment, as proved by the transwell migration assay (Fig. 5*D*) and wound healing assay (Fig. 5, *E* and *F*). Additionally, the effect of CDC20 inhibition on tumor growth was examined by subcutaneous implantation of NB cells into the groin area of NSG mice, followed by regular Apcin treatment *via* intraperitoneal injection. The results demonstrated that Apcin treatment strongly inhibited the growth of subcutaneous xenografts derived from both *MYCN*-amplified (Fig. 5, *G* and *H*) and *MYCN*-nonamplified cells (Fig. 5, *I*–*K*), with no effect on mouse body weight (Fig. 5*L*). To determine whether the phenotypic effects of Apcin were dependent on CDC20, we next compared its activity in control and CDC20-depleted NB cells. Apcin markedly suppressed colony formation and migration in control cells. CDC20 depletion itself significantly impaired these phenotypes, whereas the additional inhibitory effects of Apcin were substantially attenuated when CDC20 was already depleted (Fig. S4, *C* and *D*). These findings indicate that depletion of CDC20 reduces the phenotypic responsiveness of NB cells to Apcin. We further performed a reciprocal genetic-pharmacological experiment to determine whether ectopic CDC20 expression could counteract the effects of Apcin. Apcin markedly increased apoptosis and inhibited migration in vector-control cells. In contrast, ectopic CDC20 expression significantly attenuated Apcin-induced apoptosis and partially restored migratory capacity in both NB cell lines (Fig. S4, *E* and *F*). Together, the attenuated response to Apcin following CDC20 depletion and the partial reversal of Apcin-induced phenotypes by CDC20 overexpression provide complementary evidence that a substantial component of the antitumor activity of Apcin in NB cells is CDC20-dependent.

**Figure 5:**
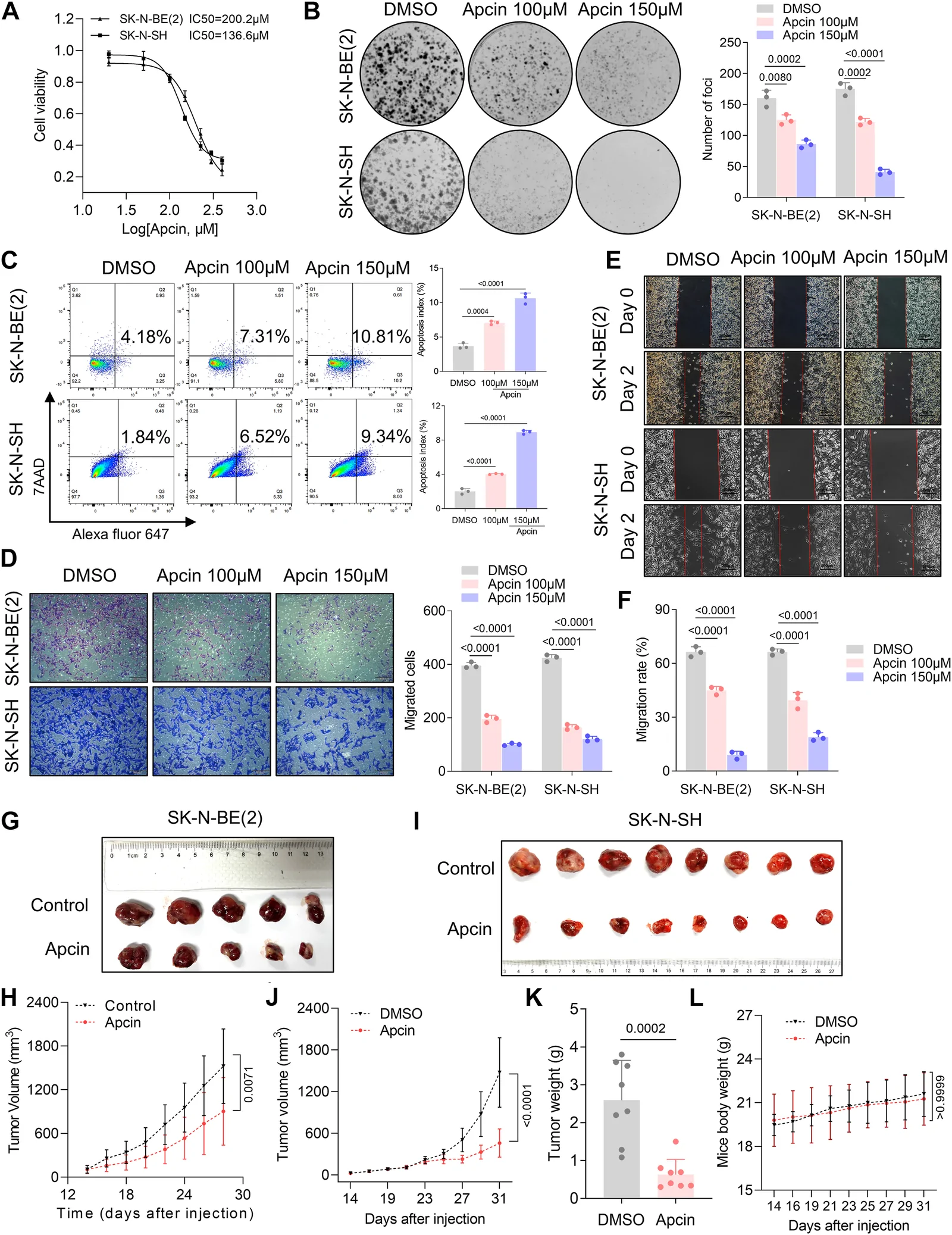
**Pharmacological inhibition of CDC20 reduces tumor growth and metastasis in NB.***A*, IC50 values of SK-N-BE(2) and SK-N-SH cells were determined by CCK-8 assay after treatment with Apcin at indicated concentrations for 48 h. Data were represented as means ± SD. *B*, Representative images of foci formation in monolayer culture in SK-N-BE(2) and SK-N-SH cells treated with Apcin at indicated concentrations for 2 weeks (*left*). The numbers of foci were calculated and depicted in the bar chart (*right*). *C*, Representative flow cytometry plots (*left*) and summary (*right*) of apoptotic cells in SK-N-BE(2) and SK-N-SH cells treated with Apcin at indicated concentrations for 24 h. *D*, Transwell migration assay was used to compare cell motility between DMSO control and Apcin-treated groups in SK-N-BE(2) and SK-N-SH cells. Representative images of migration were shown. Scale bars, 200 μm. The number of migrated cells was calculated and depicted in the bar chart. *E*, Representative images of wound healing assay in SK-N-BE(2) and SK-N-SH cells treated with Apcin at indicated concentrations for 48 h. Scale bars, 200 μm. *F*, The migration rate of cells was illustrated in the bar chart. *G* and *I*, Images of xenograft tumors induced by subcutaneous injection of SK-N-BE(2) cells (*G*) and SK-N-SH cells (*I*) into NSG mice and treated by Apcin through intraperitoneal injection at 20 mg/kg every other day for 2 weeks. *H*, Tumor growth curves were summarized in the line chart in SK-N-BE(2) cells (n = 5). *J*, Tumor growth curves were summarized in the line chart in SK-N-SH cells (n = 8). *K*, Tumor weight was summarized in the bar chart (n = 8). *L*, Mouse body weight was monitored during the intervention process. Data are presented as mean ± SD from three independent biological experiments, with individual data points shown. Statistical analyses were performed using one-way ANOVA with Dunnett's multiple comparisons test for (*B–D*) and (*F*), two-way ANOVA with Bonferroni's multiple-comparisons test for (*H*), (*J*) and (*L*), two-tailed unpaired Student's *t* test for (*K*). Exact *p* values are indicated in the figures. A *p* value < 0.05 was considered statistically significant.

Given the relatively high concentrations of Apcin required to inhibit NB cells, we next evaluated Apcin-A as an additional CDC20-targeting pharmacological approach. Apcin-A inhibited cell viability in a dose-dependent manner, with IC50 values of approximately 98 μM in SK-N-BE(2) cells and 47 μM in SK-N-SH cells (Fig. S5*A*). Consistently, Apcin-A significantly suppressed NB cell proliferation over time (Fig. S5*B*), increased apoptosis (Fig. S5*C*), and markedly reduced cell migration (Fig. S5*D*). Thus, an alternative Apcin-derived compound targeting the APC/C–CDC20 pathway reproduced the major antitumor phenotypes observed following Apcin treatment. Collectively, these results demonstrate that pharmacological inhibition of CDC20 suppresses NB cell growth and tumor progression in models representing different *MYCN* amplification statuses. Together with the genetic loss-of-function findings, these data support CDC20 as a potential therapeutic vulnerability across NB subtypes.

### GATA3 is identified as a direct transcriptional activator of CDC20

To delve deeper into the potential regulatory network, including upstream and downstream mechanisms of CDC20 in NB, we first analyzed the differentially expressed genes between the CDC20^high^ and CDC20^low^ groups. Here, we analyzed RNA-sequencing data from the NB GEO dataset (GSE45547) bioinformatically, which includes tumors across a spectrum of stages and risk groups. NB samples were ranked by CDC20 expression, and the DEGs were identified between the top 20 samples with the highest (CDC20^high^) and the bottom 20 cases with the lowest (CDC20^low^) expression. Using Limma (log2 FC ≥ 2 or ≤ −2, *p* < 0.05), 220 upregulated genes and 205 downregulated genes were found (Fig. 6*A*). The expression profile of these genes in the two groups was shown (Fig. 6*B*). For the upstream mechanisms, we mainly focus on the primary regulators of gene expression - transcription factors (TFs). To discern the TFs responsible for observed differential gene expression patterns in CDC20^high^ tumors, a computational enrichment analysis was conducted using ChEA3, a web-based TF Enrichment Analysis (TFEA) platform (19). This tool systematically prioritizes TFs by evaluating the statistical enrichment of overlaps between input gene-sets and precompiled TF-targets assembled from multiple resources such as chromatin immunoprecipitation sequencing (ChIP-seq) datasets, knock-down/overexpression studies, and regulatory pathway databases. Specifically, the 220 upregulated genes were presented to the ChEA3 tool, and the results showed that GATA3 was the most likely upstream TF that controlled the upregulation of observed altered genes in NB (Fig. 6*C*). Surprisingly, public ChIP-seq datasets in several NB cells downloaded from the JASPAR database (20) offered strong evidence that GATA3 may directly bind to the promoter region of *CDC20* (Fig. 6*D*). DepMap analysis using the online database (The Cancer Dependency Map Project at Broad Institute) confirmed that both *CDC20* and *GATA3* are essential genes in NB models (Fig. 6*E*). GATA3, a known adrenergic (ADRN) marker and oncogenic driver in NB, has been reported to positively regulate differentiation and proliferation of NB cells (21). The expression of GATA3 was also detected in CBMC and NB cell lines at both the mRNA level (Fig. S6*A*) and the protein level (Fig. S6, *B–D*). Expression data from the public datasets also supported the high expression of GATA3 in both *MYCN*-amplified and -nonamplified NB cell lines relative to normal cell lines (Fig. S6*E*) and showed an elevated tendency in *MYCN*-nonamplified tumors (Fig. S6*F*) as well as *MYCN*-nonamplified high-risk tumors (Fig. S6*G*). Also, the pro-tumorigenic and pro-metastatic roles of GATA3 in NB cells were confirmed by various *in vitro* functional experiments (Fig. S7, *A–F*). In addition, a significant positive correlation existed between *CDC20* and *GATA3* expression across multiple NB cohorts, including all NB (Fig. 6*F*) and *MYCN*-nonamplified NB (Fig. 6*G*). Collectively, these findings strongly propose that GATA3 may be the direct upstream TF regulator of CDC20.

**Figure 6:**
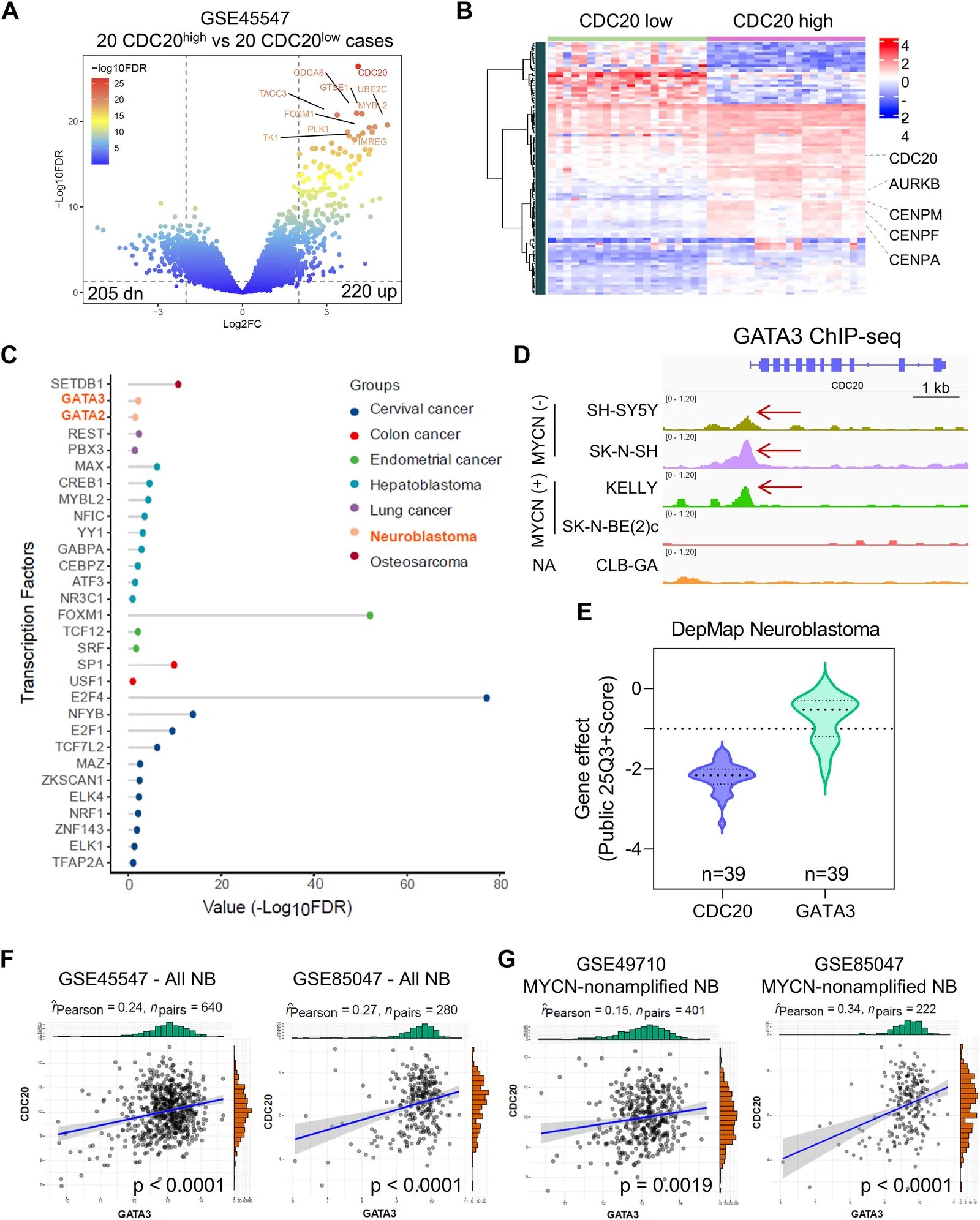
**GATA3 is identified as a direct transcriptional activator of CDC20.***A*, Volcano plot depicted the differentially expressed genes (DEGs) between the CDC20^high^ and CDC20^low^ groups by Limma R analysis (log2 FC ≥ 2 or ≤ −2, *p* < 0.05) in GSE45547 NB dataset. *B*, Heatmap plot showed the expression profile of DEGs in the CDC20^high^ and CDC20^low^ groups. *C*, Transcription factor enrichment analysis accomplished by the online ChEA3 platform based on up-regulated genes in the CDC20^high^ group identified 2 TFs, GATA3 and GATA2, as the potential upstream drivers regulating the observed altered genes in the CDC20^high^ NB. *D*, Public ChIP-seq datasets from the JASPAR database in several NB cells showed the enrichment of GATA3 at the promoter region of *CDC20*. *E*, Violin plot shows the Gene effect score of *CDC20* and *GATA3* in NB using the online DepMap database. *F* and *G*, The expression relationship between *CDC20* and *GATA3* in NB tissues and specifically *MYCN*-nonamplified NB tissues based on several GEO datasets (GSE45547, GSE85047, and GSE49710). Statistical analyses were performed using Pearson Correlation Analysis. Exact *p* values are indicated in the figures. A *p* value < 0.05 was considered statistically significant.

### GATA3 directly binds to and transcriptionally activates the *CDC20* promoter through a specific GATA3-responsive element

Given the possible connection between GATA3 and CDC20 expression, we next pinpointed their regulatory relationship and potential precise binding site. GATA3-silenced cells were generated by siRNA, and the knockdown effect was detected by qRT-PCR (Fig. 7*A*) and Western blot analysis (Fig. 7*B*). Upon GATA3 downregulation, the expression of CDC20 was potently reduced in NB cells both at the mRNA level (Fig. 7*A*) and at the protein level (Fig. 7*B*). To confirm whether the TF GATA3 could regulate the promoter activity of *CDC20*, the full-length promoter sequence of *CDC20* was introduced into a firefly-luciferase and renilla-luciferase–containing plasmid, which was then co-transfected into different NB cells together with GATA3-siRNA. Dual-luciferase reporter assay was carried out, and the result showed that the *CDC20* promoter activity was significantly decreased upon GATA3 knockdown (Fig. 7*C*), suggesting that GATA3 could directly influence the transactivation of the *CDC20* promoter. Subsequently, luciferases-carrying plasmids cloned with different truncated segments were ordered (Fig. 7*D*). Luciferase reporter assay was then repeated in NB cells transduced with different truncated mutants of the *CDC20* promoter containing plasmids. The results displayed that the segments (−1925 to −1425 and −1425 to −925) maintained consistent transactivation activity compared to the full-length of *CDC20* promoter and other truncated sequences, suggesting that this region (−1925 to −925) on the *CDC20* promoter is crucial for the transactivation of CDC20 (Fig. 7*E*). To further identify the precise binding site of GATA3 on the *CDC20* promoter region, which is responsible for its promoter activity, the prediction of the GATA3 binding motif on the promoter region of *CDC20* was performed by JASPAR software. The predicted binding motifs of GATA3 on the *CDC20* promoter were shown in Figure 7*F* and Table S2. Among the six GATA3 predicted binding sites, two (Site-1: 5'-AGATAATA-3' and Site-2: 5'-CGATAATG-3') were located between −1925 and −925 region on the *CDC20* promoter (Fig. 7*G*). Furthermore, deletion mutants of these two sites were cloned into the luciferase reporter plasmid, respectively. Upon applying site-directed mutagenesis on each binding site, we found that deletion of site-2 significantly reduced *CDC20* promoter activity in the luciferase reporter assay, while mutagenesis of site-1 showed no significance (Fig. 7*H*). Because deletion of a promoter sequence could potentially alter local promoter architecture, we further introduced site-directed nucleotide substitutions into the core site-2 GATA3-binding motif while preserving the surrounding promoter sequence. Specifically, the wild-type sequence 5′-CGATAATG-3′ was mutated to 5′-CGCTACTG-3′. Compared with the corresponding wild-type reporter, the site-2 point-mutant reporter exhibited significantly reduced luciferase activity in both SK-N-BE(2) and SK-N-SH cells (Fig. 7*I*), confirming the functional importance of this motif for *CDC20* promoter activity. Finally, ChIP-qPCR demonstrated significant enrichment of GATA3 specifically at the *CDC20* promoter region encompassing site 2, whereas no significant enrichment was detected at the other tested promoter regions in SK-N-BE(2) cells (Fig. 7*J*). Altogether, these results clearly demonstrated that GATA3 could directly bind to *CDC20* promoter at the −1425 to −925 region, with the specific binding site (the consensus DNA sequence 5'-CGATAATG-3') at the −1392 to −1385 region. As a result, CDC20 expression could be transcriptionally regulated by GATA3 in NB.

**Figure 7:**
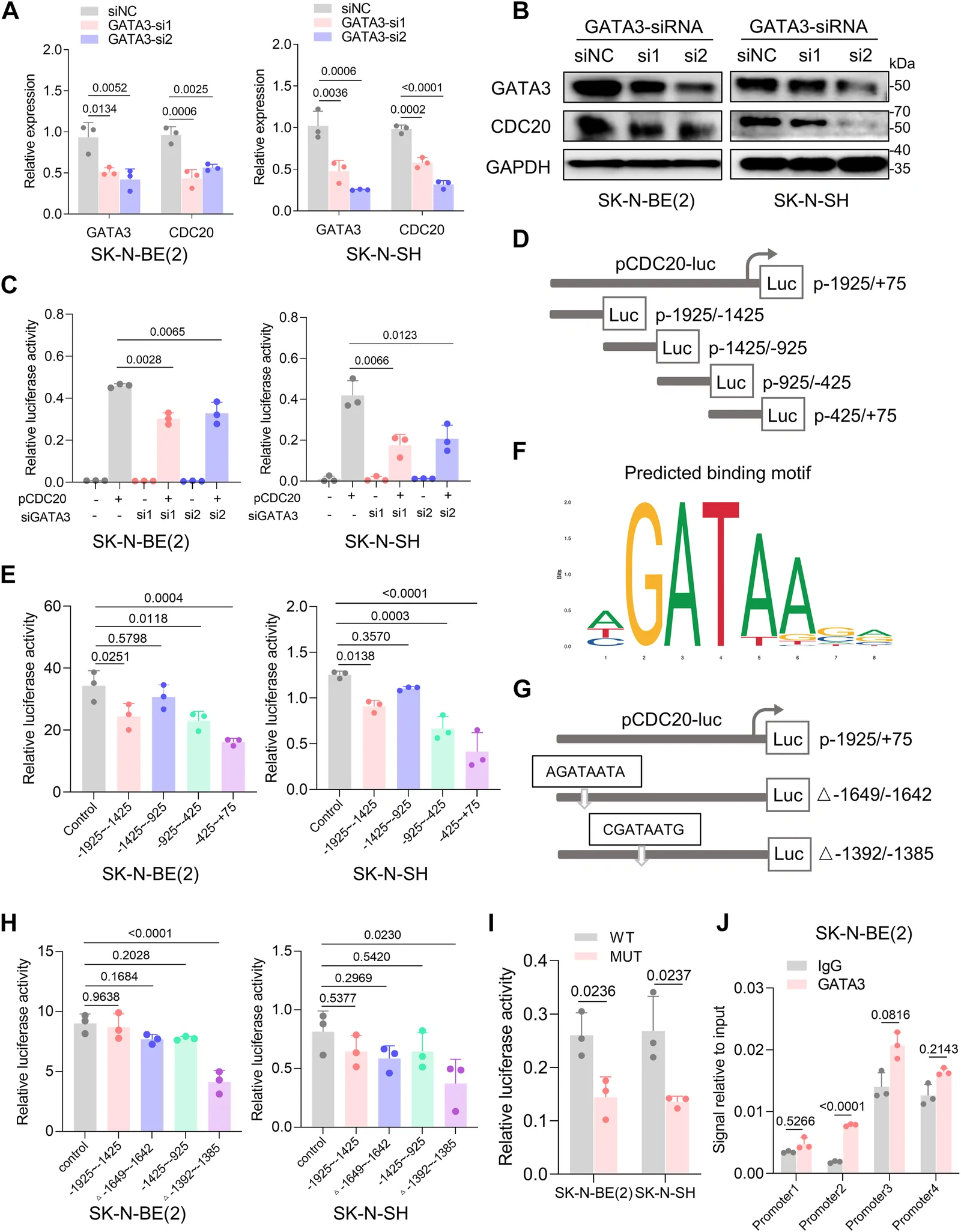
**GATA3 directly binds to and transcriptionally activates the *CDC20* promoter through a specific GATA3-responsive element.***A*, qRT-PCR assay showing the effect of *GATA3* knockdown on *CDC20* mRNA levels in SK-N-BE(2) and SK-N-SH cells. *B*, Western blot analysis showing the effect of GATA3 knockdown on CDC20 protein levels in SK-N-BE(2) and SK-N-SH cells. GAPDH was included as the loading control. *C*, Relative luciferase activity in the full length of *CDC20* promoter (p*CDC20*) indicated by dual-luciferase reporter assay in SK-N-BE(2) and SK-N-SH cells co-transfected with luciferase reporter plasmid and siNC/siGATA3. *D*, Schematic representation of full-length and truncated *CDC20* promoter sequence. *E*, Relative luciferase activity in each of the truncated mutants of *CDC20* promoter, the full length of *CDC20* promoter (Control) indicated by dual-luciferase reporter assay. *F*, Prediction of the GATA3 binding motif on the promoter of *CDC20* by JASPAR revealed six potential binding sites. *G*, Schematic representation of site-directed deletion mutants of *CDC20* promoter sequence. Site-1: 5-AGATAATA-3 and Site-2: 5-CGATAATG-3. *H*, Relative luciferase activity after deletion mutagenesis of the candidate binding sites indicated by dual-luciferase reporter assay. Deletion of Site-2 significantly reduced *CDC20* promoter activity. *I*, Site-directed point-mutational analysis of Site-2. The wild-type GATA3-binding sequence (WT, 5′-CGATAATG-3′) was changed to 5′-CGCTACTG-3′ in the point-mutant construct (MUT) without altering the length of the promoter sequence. The activities of the WT and Site-2 MUT *CDC20* promoter reporters were compared in parental SK-N-BE(2) and SK-N-SH cells using dual-luciferase reporter assays. *J*, The GATA3 binding activity was further corroborated using a ChIP-qPCR assay performed in SK-N-BE(2) cells. Data are presented as mean ± SD from three independent biological experiments, with individual data points shown. Statistical analyses were performed using one-way ANOVA with Dunnett's multiple comparisons test for (*A*), (*C*), (*E*) and (*H*), two-tailed unpaired Student's *t* test for (*I* and *J*). Exact *p* values are indicated in the figures. A *p* value < 0.05 was considered statistically significant.

### Genetic epistasis establishes CDC20 as a functional downstream effector of GATA3 in NB cells

To establish the functional hierarchy between GATA3 and CDC20, we first detected whether CDC20 could regulate GATA3 expression and found that CDC20 knockdown (Fig. S8, *A* and *B*) or overexpression (Fig. S8*C*) has no effect on GATA3 expression, supporting a unidirectional regulatory relationship in which GATA3 functions upstream of CDC20. We next performed complementary genetic epistasis experiments in SK-N-BE(2) and SK-N-AS cells to determine whether CDC20 mediates the tumor-promoting effects of GATA3. First, CDC20 was depleted in GATA3-overexpressing cells to determine whether CDC20 is required for GATA3-driven phenotypes. qRT-PCR and Western blot analyses confirmed that GATA3 overexpression increased CDC20 expression, whereas concomitant CDC20 knockdown effectively reduced CDC20 expression without substantially affecting the ectopic expression of GATA3 (Fig. 8, *A* and *B*). Functionally, GATA3 overexpression markedly enhanced colony formation and migration in both NB cell lines, whereas CDC20 depletion significantly abrogated these GATA3-induced effects (Fig. 8, *C* and *D*). These findings indicate that CDC20 is required for the pro-tumorigenic effects of GATA3. We then performed the reciprocal rescue experiment to further establish the functional relationship between GATA3 and CDC20. GATA3 was depleted and CDC20 was subsequently restored by ectopic expression. qRT-PCR and Western blot analyses confirmed efficient GATA3 knockdown and restoration of CDC20 expression in both cell lines (Fig. 8, *E* and *F*). GATA3 depletion markedly suppressed colony-forming ability and cell migration, whereas restoration of CDC20 significantly rescued both phenotypes (Fig. 8, *G* and *H*). Furthermore, GATA3 depletion increased apoptosis in both SK-N-BE(2) and SK-N-AS cells, and ectopic CDC20 expression significantly attenuated this increase in apoptosis (Fig. 8*I*). Together, these reciprocal genetic epistasis experiments demonstrate that CDC20 functions downstream of GATA3 and mediates, at least in part, the effects of GATA3 on NB cell growth, migration, and survival.

**Figure 8:**
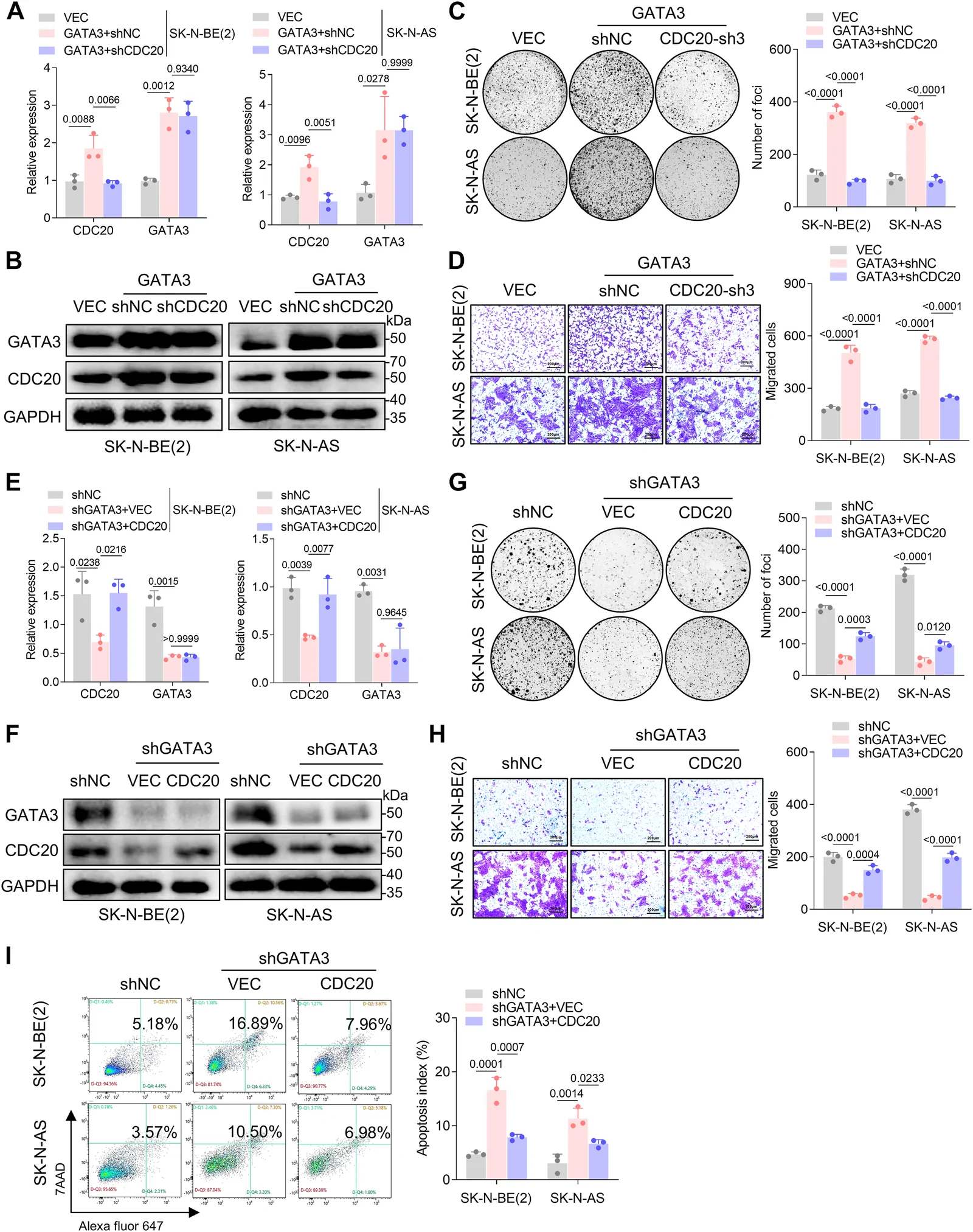
**Genetic epistasis establishes CDC20 as a functional downstream effector of GATA3 in neuroblastoma cells.***A*, qRT-PCR analysis of *CDC20* and *GATA3* expression in SK-N-BE(2) and SK-N-AS cells transduced with control vector (VEC), GATA3 together with control shRNA (GATA3 + shNC), or GATA3 together with CDC20-targeting shRNA (GATA3 + shCDC20). *B*, Western blot analysis of GATA3 and CDC20 protein expression in the indicated cells. GAPDH was used as a loading control. *C*, Representative images and quantification of foci formation in monolayer culture in the indicated cells. *D*, Representative images and quantification of transwell migration assays in the indicated cells. Scale bars, 200 μm. *E*, qRT-PCR analysis of *CDC20* and *GATA3* expression in SK-N-BE(2) and SK-N-AS cells transduced with control shRNA (shNC), GATA3-targeting shRNA together with control vector (shGATA3 + VEC), or GATA3-targeting shRNA together with ectopic CDC20 (shGATA3 + CDC20). *F*, Western blot analysis confirming GATA3 depletion and restoration of CDC20 protein expression in the indicated cells. GAPDH was used as a loading control. *G*, Representative images and quantification of foci formation assays following GATA3 depletion and CDC20 restoration in SK-N-BE(2) and SK-N-AS cells. *H*, Representative images and quantification of transwell migration assays in the indicated cells. Scale bars, 200 μm. *I*, Representative flow cytometry plots and quantification of apoptosis in SK-N-BE(2) and SK-N-AS cells following GATA3 depletion and CDC20 restoration. Data are presented as mean ± SD from three independent biological experiments, with individual data points shown. Statistical analyses were performed using one-way ANOVA with Tukey's multiple comparisons test for (*A*), (*C–E*) and (*G–I*). Exact *p* values are indicated in the figures. A *p* value < 0.05 was considered statistically significant.

### CDC20 promotes EMT-like properties and apoptotic resistance in neuroblastoma

To explore the downstream mechanisms of CDC20 in NB, transcriptomic analysis between CDC20^high^ and CDC20^low^ NB tissues was performed. In brief, the 20 NB cases with the highest *CDC20* expression and the 20 NB cases with the lowest *CDC20* expression in the GSE45547 datasets were selected and 220 upregulated genes in CDC20^high^ tumors were identified as shown in Figure 6*A*. GSEA on these upregulated genes was performed. The hallmarks enrichment demonstrated that this cluster of genes was meticulously related to TNFα signaling *via* NF-κB, apoptosis, and epithelial–mesenchymal transition (EMT) process (Fig. 9*A*). The KEGG enrichment revealed DNA replication, epithelial cell signaling and focal adhesion-related pathways in *CDC20* high expression NB group (Fig. S9*A*). We also performed bulk RNA-seq in SK-N-SH cells transfected with CDC20-shNC or sh1. By using Limma (log2 FC ≥ 1 or ≤ −1, *p* < 0.05), 220 up-regulated genes and 342 downregulated genes were found (Fig. 9*B*). GSEA on the 342 downregulated genes revealed the consistent enrichment of pathways including apoptosis, migration and cell adhesion (Figs. 9*C* and S9, *B* and *C*). These data further support the role of CDC20 in facilitating tumor growth and metastasis in NB. Consistent with this, next, to investigate whether the pro-metastatic ability of CDC20 was triggered by EMT, the expression of multiple epithelial and mesenchymal markers was detected both at the mRNA level by qRT-PCR (Fig. S9*D*) and protein level by Western blot (Fig. 9*D*) in NB cells upon CDC20 knockdown. The results revealed that CDC20 silencing could increase epithelial marker E-cadherin and eliminate mesenchymal markers such as N-cadherin, Vimentin, Snail, Slug, and ZEB1, indicating that CDC20 could promote EMT in NB cells. IHC staining of the three representative markers in mouse tumor tissues from the liver-metastasis site confirmed these changes (Fig. 9*E*). Given that the upstream regulator of CDC20, GATA3, is a known adrenergic marker of NB (22), we also analyzed adrenergic/mesenchymal transcription factors upon CDC20 knockdown but found no significant changes (Fig. S9*E*), suggesting CDC20's effects on plasticity are distinct from core lineage regulation. Additionally, since the ratio of Bax to Bcl-2 dictates the cellular susceptibility to apoptosis (23), we used a Western blot method to detect the altered expression of Bax and Bcl-2. The results indicated that upon CDC20 downregulation, the Bax/Bcl-2 ratio was significantly upregulated, leading to higher susceptibility to apoptosis and augmented cell apoptosis (Fig. 9*F*). Beyond the Bax/Bcl-2 ratio, we investigated whether CDC20 influences a broader spectrum of apoptosis regulators. The qRT-PCR analysis of additional key genes revealed that CDC20 knockdown also led to a significant decrease in the mRNA expression of the apoptosis-related genes *BCL2L10* and the executioner caspase *CASP3* (Figs. 9*G* and S9*F*). The downregulation of *BCL2L10* *mRNA* is consistent with a shift toward a pro-apoptotic state. While the observed decrease in *CASP3* mRNA may indicate complex feedback within the apoptosis pathway, these changes collectively underscore that CDC20 modulates a network of apoptosis-associated genes. Together with the functional data on the Bax/Bcl-2 axis, this positions CDC20 as a multi-faceted regulator of apoptotic resistance in NB, with the Bax/Bcl-2 axis representing a central, functionally validated component. These findings suggest that CDC20 promotes NB progression by regulating the EMT process and Bax/Bcl-2 pathways.

**Figure 9:**
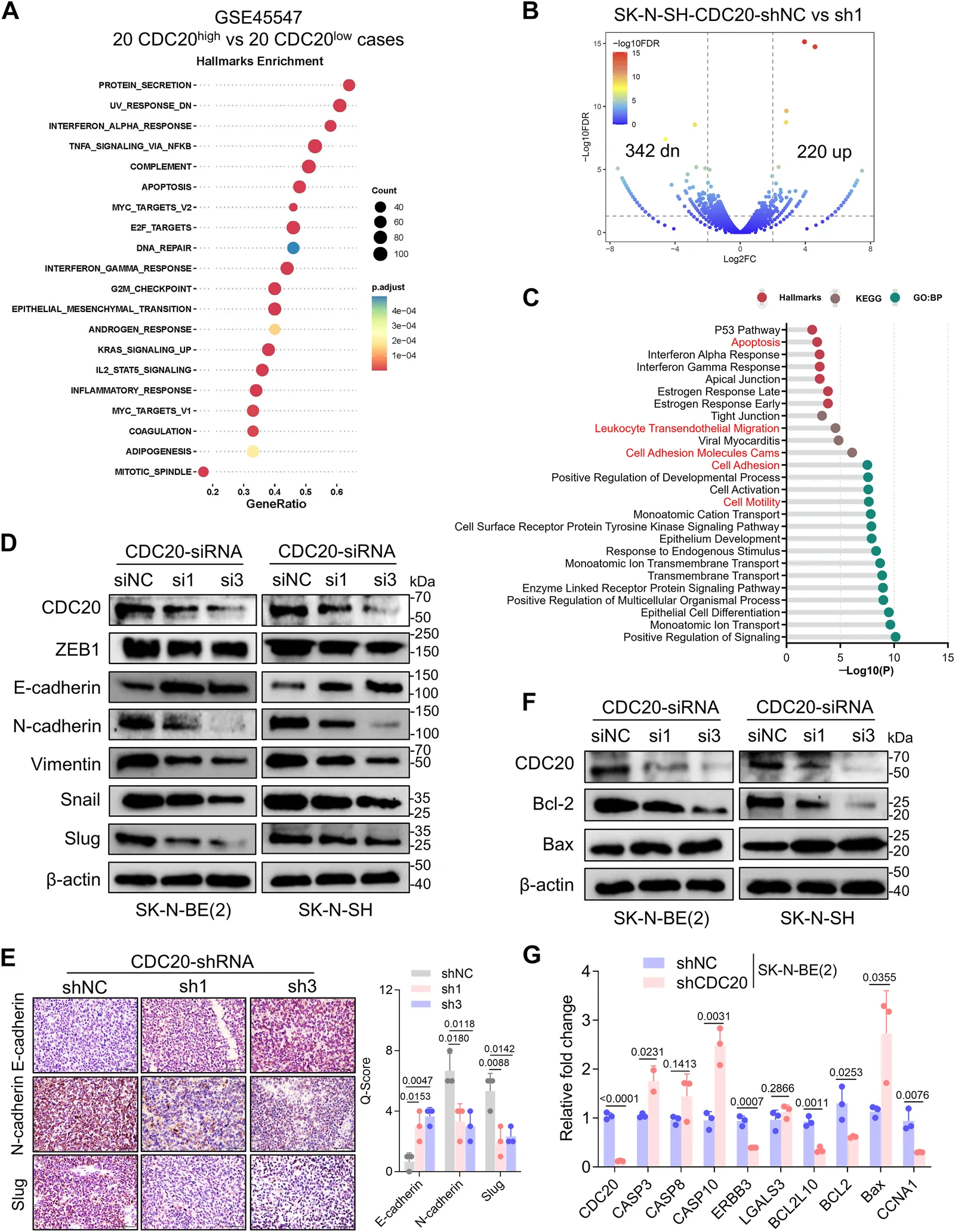
**CDC20 promotes EMT-like changes and apoptotic resistance in neuroblastoma cells.***A*, The hallmarks enrichment analysis based on 220 up-regulated genes in CDC20^high^ NB tissues in contrast to CDC20^low^ NB tissues. *B*, Bulk RNA-seq was performed in SK-N-SH cells transfected with shNC or sh1. Volcano plot depicted the differentially expressed genes (DEGs) between the CDC20-shNC and CDC20-sh1 groups by Limma R analysis (log2 FC ≥ 1 or ≤ −1, *p* < 0.05). *C*, GSEA analysis showing the enrichment of hallmarks, KEGG and GO-BP pathways based on 342 down-regulated genes upon CDC20 knockdown. *D*, Western blot analysis showing the effect of CDC20 knockdown on the protein levels of multiple epithelial and mesenchymal markers in SK-N-BE(2) and SK-N-SH cells. *E*, Representative images of IHC staining of the three indicated markers in mouse NB tumor tissues from the liver-metastasis site. The semi-quantitative Q-score was shown in the bar chart. Scale bars, 50 μm. *F*, Western blot analysis showing the effect of CDC20 knockdown on the protein levels of apoptosis-associated axis, Bax and Bcl-2. *G*, qRT-PCR assay showing the effect of CDC20 knockdown on the mRNA levels of multiple apoptosis-related genes in SK-N-BE(2) cells. Data are presented as mean ± SD from three independent biological experiments, with individual data points shown. Statistical analyses were performed using one-way ANOVA with Dunnett's multiple comparisons test for (*E*) and two-tailed unpaired Student's *t* test for (*G*). Exact *p* values are indicated in the figures. A *p* value < 0.05 was considered statistically significant.

Given that CDC20 regulates EMT- and apoptosis-associated proteins, we next examined whether its upstream regulator GATA3 produces corresponding molecular changes. GATA3 depletion in SK-N-BE(2) and SK-N-AS cells reduced the expression of N-cadherin, Slug, and Bcl-2 (Fig. S9, *G* and *H*). GATA3 knockdown also decreased ZEB1 and Vimentin expression while increasing Bax expression. These findings indicate that GATA3 and CDC20 regulate concordant molecular programs associated with EMT-like properties and apoptotic resistance in NB cells.

## Discussion

Neuroblastoma is a complex pediatric cancer that presents significant challenges in both diagnosis and treatment. While *MYCN* amplification is a hallmark of high-risk NB, the molecular drivers of aggressive phenotypes in *MYCN*-nonamplified high-risk cases are poorly understood. This highlights the need for a broader investigation of the genetic and transcriptomic molecular landscape in NB beyond *MYCN* amplification. A recent study revealed that *MYCN*-nonamplified neuroblastomas exhibited transcriptional heterogeneity and could be stratified into three distinct molecular subtypes with divergent clinical outcomes (24). And the Subgroup_2 with the poorest outcomes exhibited an overexpression of Aurora Kinase A (AURKA), demonstrating that AURKA may be a potentially oncogenic driver of *MYCN*-nonamplified NB (24). Furthermore, the genomic landscapes of *MYCN*-nonamplified NB have been studied through whole-exome sequencing (WES) and whole transcriptome sequencing (WTS). Intriguingly, high-risk tumors exhibited enriched genomic alterations in metabolic pathways, underscoring the existence of genomic heterogeneity within *MYCN*-nonamplified cases (25). This study identifies and characterizes the oncogenic role of CDC20 in neuroblastoma, with a focus on addressing the unmet need for drivers in the *MYCN*-nonamplified high-risk subgroup. While our initial bioinformatic screen targeted this subgroup, functional validation revealed that CDC20's oncogenic role extends across NB subtypes. However, its clinical utility as a biomarker is context-dependent: in *MYCN*-nonamplified patients, high CDC20 expression is a powerful indicator of high-risk disease, metastasis, and poor OS and DFS. In *MYCN*-amplified patients, who already have a poor prognosis, CDC20 adds value primarily by predicting worse DFS, likely reflecting metastatic potential. This nuanced prognostic signature underscores CDC20's particular relevance for risk stratification within the heterogeneous *MYCN*-nonamplified population. The inclusion of both *MYCN*-amplified and *MYCN*-nonamplified models therefore allowed us to distinguish the subgroup-specific prognostic utility of CDC20 from its broader oncogenic activity. These observations suggest that CDC20 may represent a common functional vulnerability across molecularly distinct NB subtypes, while its prognostic relevance may be particularly useful for refining risk assessment among *MYCN*-nonamplified patients. Future studies in larger, prospectively characterized cohorts will be required to determine whether CDC20 can provide prognostic information independent of established clinical and molecular risk factors.

Pan-cancer analyses revealed ubiquitous CDC20 overexpression across malignancies, correlating with advanced disease stage, metastatic propensity, and inferior overall survival (26). These parallels underscore the oncogenic role of CDC20 across cancers, supporting its potential as a therapeutic target. The therapeutic potential of targeting CDC20 was substantiated by Apcin-mediated inhibition, which attenuated tumor growth and metastasis in preclinical models (26, 27, 28). In our study, therapeutic targeting of CDC20 with Apcin showed efficacy in preclinical models. The relatively high *in vitro* IC50 of Apcin required in our NB models warrants caution, as off-target effects at such concentrations cannot be ruled out. To address this, we integrated biochemical and genetic approaches. Apcin induced accumulation of Cyclin B1 and securin, consistent with APC/C–CDC20 inhibition. Importantly, CDC20 depletion reduced Apcin sensitivity, whereas CDC20 overexpression partially rescued Apcin-induced apoptosis and migration defects, supporting a CDC20-dependent mechanism. In addition, the alternative compound Apcin-A showed improved potency and recapitulated the major anti-tumor effects of Apcin. Together with genetic loss-of-function data, these findings support pharmacological targeting of CDC20 in NB, although compound specificity remains incomplete and more potent, selective inhibitors are needed for translational development.

The consistent effects of genetic CDC20 depletion and pharmacological CDC20 inhibition in models with different *MYCN* amplification statuses suggest that the oncogenic dependency on CDC20 is not restricted to *MYCN*-nonamplified NB. Thus, CDC20 may represent a therapeutic vulnerability across NB molecular subtypes, whereas its particular relevance in *MYCN*-nonamplified disease lies in its potential utility for additional prognostic and risk stratification. Nevertheless, given the relatively high concentrations of Apcin required to inhibit NB cells, further development and evaluation of more potent, and selective CDC20-targeting approaches will be necessary before this strategy can be translated therapeutically.

For the upstream mechanism, we identified GATA3 as the direct transcriptional activator of CDC20, a previously uncharacterized regulatory relationship in NB. While GATA3 is a core component of the adrenergic state often linked to *MYCN*-amplified tumors (21, 29, 30), our analysis shows it is also highly expressed in a subset of aggressive *MYCN*-nonamplified cases. In this context, GATA3 appears to activate alternative pro-tumorigenic programs, with CDC20 being a prime example. This expands GATA3's functional repertoire beyond lineage maintenance to include direct regulation of cell cycle and plasticity regulators, highlighting the multifaceted role of lineage factors in cancer progression. This provides a mechanistic hypothesis for how adrenergic tumors might co-opt a differentiation factor to fuel progression, highlighting the importance of epigenetic and transcriptional drivers in *MYCN*-independent tumors. Further validation in other cancers could clarify whether this axis is NB-specific or a broader oncogenic mechanism.

The multifaceted characters of CDC20 and its downstream mechanisms have been well explored and reported. As an evolutionarily conserved coactivator of the anaphase-promoting complex/cyclosome (APC/C), CDC20 serves as a critical mitotic checkpoint regulator by orchestrating the metaphase-to-anaphase transition through timed degradation of securin and cyclin B. This ensures faithful chromosome segregation and genomic fidelity. However, cancers could hijack this process to promote mitotic progression and accelerate malignant proliferation. The aberrant overexpression of CDC20 disrupts mitotic fidelity, induces chromosomal instability, and fosters tumorigenic evolution (31). In this study, CDC20 facilitated cell proliferation in NB, which may be attributed to its canonical role as an APC/C coactivator in mitotic exit. Additionally, emerging evidence suggests that CDC20 may regulate apoptotic signaling and EMT beyond its canonical mitotic function. For example, CDC20 has been reported to promote ubiquitination-mediated degradation of pro-apoptotic regulators such as Bim (32) and pro-metastatic markers such as FOXO1 (33), thereby attenuating apoptosis and metastasis in multiple cancer contexts. The non-canonical role of CDC20 in apoptotic resistance *via* Bax/Bcl-2 ratio suppression in NB also aligns with its role in other cancers (34, 35). The Bax/Bcl-2 axis is a pivotal determinant of apoptosis in cancer, implicated in tumorigenesis, metastasis, and therapeutic response (36, 37). In our study, CDC20 promotes NB progression through dual mechanisms: the Bax/Bcl-2 imbalance and EMT induction. These findings align with emerging evidence linking CDC20 to metastatic programs in other cancers but are newly established in NB (38, 39). Although the precise substrates of CDC20 in neuroblastoma remain to be fully elucidated, these findings provide a plausible mechanistic framework through which CDC20 upregulation may contribute to apoptosis resistance and metastasis in this disease.

In conclusion, our study integrates multi-omics data, functional assays, and clinical validation to delineate the role of CDC20 in NB oncogenicity. We establish CDC20 as a GATA3-regulated oncoprotein that drives NB progression through EMT induction and apoptotic resistance, supporting CDC20 as a potential therapeutic vulnerability across NB subtypes while highlighting its particular potential for improving risk stratification in *MYCN*-nonamplified disease. Further studies will be required to establish the clinical utility of CDC20-based stratification and to develop more potent and selective approaches for therapeutic CDC20 inhibition.

## Experimental procedures

### NB cell lines and clinical specimens

The human cell lines, including cord blood mononuclear cell (CBMC) and immortalized NB cell lines like SH-SY5Y, SK-N-SH, SK-N-BE(2), IMR32, and SK-N-AS were purchased from COBIOER BIOSCIENCES. All cell lines were confirmed to be mycoplasma-free with authenticated STR profiles. SK-N-SH and IMR32 cells were maintained in Minimum Essential Medium (MEM; Gibco, USA) with 10% fetal bovine serum (FBS, HyClone, USA), 1% Non-Essential Amino Acids (NEAA; Gibco) and 1% Sodium pyruvate (Gibco, USA). SK-N-AS was cultured in Dulbecco's Modified Eagle Medium (DMEM; Gibco) supplemented with 10% FBS and 1% NEAA. SK-N-BE(2) and SH-SY5Y were cultured in a mixture of MEM and Ham's F-12 Nutrient Mixture (F-12; Gibco) (1:1) with 10% FBS, 1% NEAA and 1% Sodium pyruvate. All culture media contained 1% penicillin/streptomycin. Cell cultures were maintained at 37 °C in a 5% CO_2_ humidified atmosphere. This study utilized 98 neuroblastoma (NB) specimens collected from patients treated at Sun Yat-sen University Cancer Center (SYSUCC). Written informed consent was obtained from all participants for the research use of clinical samples. This clinical study was conducted in accordance with the Declaration of Helsinki principles and the protocol and sample usage were approved by the Committee for Ethical Review of Research Involving Human Subjects at Sun Yat-sen University Cancer Center (SYSUCC).

### Antibodies and reagents

The following primary antibodies were used: β-actin (1:1000, P60709, Cell Signaling Technology), GAPDH (1:1000, AC001, Abclonal), CDC20 (1:1000, Q105, Bioworld Technology), GATA3 (1:1000, MA1-028, Invitrogen), N-cadherin (1:1000, ab207608, Abcam), E-cadherin (1:1000, ab231303, Abcam), Vimentin (1:1000, ab8978, Abcam), Snail (1:1000, ab216347, Abcam), Slug (1:1000, ab302780, Abcam, USA), ZEB1 (1:1000, A21794, Abclonal), Bcl-2 (1:1000, A19693, Abclonal, China), Bax (1:1000, A19684, Abclonal), Cyclin B1 (1:1000, AF6168, Affinity), Securin (1:1000, YP-mAb-00502, UpingBio). The CDC20 inhibitor, Apcin, was purchased from TargetMol Sciences (Cat. No. T8561). The CDC20 inhibitor, Apcin-A, was purchased from MCE (Cat. No. HY-130841).

### Plasmids, oligos and transfection

CDC20 shRNA plasmids (pLKO.1-EGFP-Puro) were obtained from Dahong (Cat. No. SHNM_001255), GATA3 shRNA plasmids (Cat. No. HSH007165-LVRU6H), CDC20 (Cat. No. EX-B0269-Lv105) and GATA3 (Cat. No. EX-Z3102-Lv151) full-length containing plasmids were obtained from the GeneCopoeia. The lentiviral transfection procedure was performed following the manufacturer's protocol. The oligos ordered from GenePharma were as follows: negative control siRNA (siNC) or siRNAs targeting CDC20 (siCDC20) or siRNAs targeting GATA3 (siGATA3). The plasmids used for the luciferase reporter assay were purchased from GeneCopoeia: the luciferase reporter plasmids containing the full-length CDC20 promoter and its truncated plasmids, as well as site-directed mutants of the CDC20 promoter (pEZX-PL01). Lentivirus was generated by transfecting 293FT cells with viral plasmids. NB cells were infected with viral supernatants. The siRNA products were transiently transfected into NB cells. All oligo sequences were available in Table S3 (Supporting Information).

### qRT-PCR and Western blotting

RNA was extracted using TRIzol (Invitrogen), followed by cDNA synthesis (TaKaRa High Fidelity kit). For quantitative analysis, qRT-PCR was executed using SYBR Green PCR Kit (Dongsheng Biotech) and the primer pairs listed in Table S4 (Supporting Information). Conventional RT-PCR was conducted with DreamTaq DNA polymerase (Thermo Fisher Scientific). Gene expression at the protein level was examined by Western blot analysis according to previously published methodology (40). Antibody specificity was assessed based on the expected molecular weight and, where applicable, validated by the expected changes in protein abundance following shRNA-mediated knockdown or ectopic overexpression of the corresponding target.

### Cell viability and proliferation assays

Cell viability was quantified using a CCK-8 kit (APE x BIO) following the manufacturer's instructions. The clonogenic potential of tumor cells was assessed through colony formation assays, measuring their ability to proliferate under anchorage-dependent conditions. Briefly, 3 × 10^3^ cells were seeded in a 6-well plate. Different concentrations of the CDC20 inhibitor Apcin or Apcin-A and DMSO were supplemented. After 14 days, viable colonies were fixed with methanol, stained using 0.5% crystal violet solution, and quantified by manual counting.

### Wound healing and cell migration assays

Cells were suspended and seeded into 12-well plates with scratch plug-in components (ibidi), and cultured in 0.5% FBS medium overnight. The scratch plug-in components were removed and cell debris was washed out with phosphate-buffered saline (PBS). Different concentrations of the CDC20 inhibitor Apcin or Apcin-A and DMSO were supplemented. Baseline images of the scratch were captured using a phase-contrast microscope. After 24 to 48 h of standard incubation to allow cell migration, the same scratch regions at defined intervals were re-imaged. Quantification of the scratch area (Migration rate) was measured using image analysis software ImageJ. For the migration assay, transwell inserts (8 μm pore; Corning) were utilized. Following 24 h serum starvation for synchronization, cells were seeded in serum-free DMEM in the upper chamber, with 10% FBS-containing medium as a chemoattractant in the lower chamber. After 24 h standard incubation, migrated cells were fixed with paraformaldehyde, stained with crystal violet, and then photographed using a microscope. The quantification of migrated cells was performed using ImageJ software.

### Apoptosis assay

The cell apoptosis was assessed by Annexin V staining (4A Biotech), according to the manufacturer's protocols. Different concentrations of the CDC20 inhibitor Apcin or Apcin-A and DMSO were supplemented to the culture medium. Briefly, 5 × 10^5^ cells per well were suspended in 15 ml tubes. Cells were then stained with Annexin V-Alexa Fluor 647 and 7-AAD dyes in 1 × binding buffer provided by the kit after washing with PBS twice. Incubation at room temperature in the dark for 15 min was required for thorough staining, and flow cytometry was applied to detect apoptotic cells with Annexin-V positive, which was calculated as the apoptotic index.

### *In vivo* mouse experiments

The mice were housed in a pathogen-free laboratory animal unit at the Sun Yat-sen University (SYSU). All animal experiments were conducted in accordance with protocols approved by the Sun Yat-Sen University Cancer Center Institutional Animal Care and Use Committee. For tumorigenicity assessment, 5 × 10^6^ NB cells were subcutaneously injected into the back region of 4-week-old NSG or NOD-SCID mice. Tumor growth kinetics were monitored by weekly volumetric measurements (0.5 × length × width^2^). Following a period of 4 to 5 weeks, the mice were euthanized, and the subcutaneous tumors were harvested and imaged. For metastasis assessment, 2 × 10^6^ NB cells labelled with luciferase were injected into the tail vein of 4-week-old NSG mice. Tumor incidence and tumor growth were monitored weekly using the IVIS Spectrum imaging system (PerkinElmer) following intraperitoneal administration of 100 mg/kg D-luciferin (Promega) into the mice under isoflurane anesthesia. At the study endpoint, bioluminescence images of mice were acquired prior to euthanasia, followed by liver excision for *ex vivo* analysis. Metastatic burden was quantified by counting surface tumor nodules on hepatic tissue. Then the livers were embedded and subjected to further histological and IHC experiments. For the *in vivo* drug treatment animal model, after construction of the subcutaneous tumorigenicity model as described earlier, the CDC20 inhibitor (Apcin) was utilized by intraperitoneal injection at 20 mg/kg every other day for 2 weeks, a dosing regimen previously shown to be effective and well-tolerated in preclinical xenograft models (28, 41), starting from the 14th day. Tumor volumes and mouse body weights were monitored every 2 days and calculated by the same formula. Following a period of 4 to 5 weeks, the mice were euthanized, and the subcutaneous tumors were harvested and imaged. For all subcutaneous tumor models, mice were euthanized when tumors reached the experimental endpoint (diameter > 20 mm), or earlier upon meeting predefined humane endpoints, including severe weight loss (> 20% of initial weight), tumor ulceration/necrosis, or impaired ambulation and feeding.

### H&E and IHC staining

The excised liver tissues were fixed, dehydrated and embedded into paraffin blocks. Serial sections (4–6 μm thickness) were cut using a microtome and mounted on glass slides. The section slides were then stained with H&E for visualization of liver metastasis. For the IHC staining, clinical NB specimen slides were incubated with mouse anti-human CDC20 (Abcam) primary antibody as previously described (42). Mouse metastatic liver sections were subjected to E-cadherin, N-cadherin, and Slug primary antibodies for the detection of epithelial-to-mesenchymal transition. IHC images were acquired using a digital pathology slide scanner (KF-PRO-040-HI). The staining results were semi-quantitatively assessed by evaluating both staining intensity and the percentage of positive tumor cells. Intensity was graded as: 0 (negative), 1 (weak), 2 (moderate), or 3 (strong). The proportion of stained cells was categorized as: 0 (<25%), 1 (25–50%), 2 (50–75%), or 3 (75–100%). A combined Q-Score (intensity × percentage) was calculated, with scores 0 to 4 considered as low expression and 5 to 9 as high expression.

### Immunofluorescence staining

Cells grown on glass-bottom culture dishes (Nest Scientific) were fixed with 4% paraformaldehyde (PFA, Sigma-Aldrich), permeabilized with 0.1% Triton X-100 for 5 min, and washed with PBS. Following blocking with 5% BSA for 1 h at room temperature, primary antibody incubation was conducted overnight at 4 °C in 5% BSA solution. After three PBS washes, cells were subsequently stained with a fluorescence-labeled secondary antibody (Alexa Fluor 594-conjugated antibody, FuDe, China) for 1 h at room temperature. DAPI dye (Beyotime, Shanghai, China) was used for cell nucleus staining. After cell mounting, fluorescence images were acquired with a Zeiss LSM 800 confocal system.

### Chromatin immunoprecipitation (ChIP)-qPCR assay

The SimpleChIP Enzymatic Chromatin IP Kit (Magnetic Beads) (CST#9003) was used to extract chromatin after lysis and sonication from formaldehyde-treated SK-N-BE(2) cells with cross-linked DNA and the binding protein on chromatin. 30 μl of pretreated Protein A/G agarose beads and anti-GATA3 antibody or control Rabbit IgG (CST#2729) antibody were added to the lysate and incubated overnight at 4 °C, and the precipitated DNA was collected. The extracted chromatin DNA was used as the template for qPCR based on specific primers (listed in Table S5) to detect the binding of GATA3 at different truncated promoter regions of *CDC20*.

### Dual-luciferase reporter assays

The Dual Luciferase Reporter Assay Kit purchased from Promega (E1910) was used according to the manufacturer's directions. In brief, the full-length or different truncated sequences of the *CDC20* promoter or site-directed mutants of the *CDC20* promoter were cloned into the pEZX-PL01 control vector containing firefly luciferase as well as Renilla luciferase. The reporter plasmids (GeneCopoeia) were transfected into SK-N-BE(2) or SK-N-SH cells in 12-well plates, respectively. Two predicted GATA3-binding sites (−1649 to −1642 and −1392 to −1385) were analyzed. In addition to deletion constructs, a Site-2 point mutant (5′-CGATAATG-3′ to 5′-CGCTACTG-3′) was generated to preserve promoter context. After incubation for 24 to 48 h, cell lysis were collected, and luciferase assay substrate (Luciferase Assay Reagent II) was added. Luciferase activity was assessed using a dual-reporter system: firefly luciferase measurement followed by Renilla luciferase after signal quenching. Data were normalized as firefly/renilla ratios to control for transfection variation and cell viability.

### RNA sequencing and differential expression analysis

Total RNA was extracted from SK-N-SH cells stably expressing shNC or shCDC20 and subjected to bulk RNA sequencing. After quality control, sequencing reads were processed, aligned to the human reference genome, and quantified for gene expression. Differentially expressed genes between shNC and shCDC20 groups were identified using Limma with the indicated thresholds. Pathway enrichment was evaluated by GSEA. The RNA-seq dataset has been deposited in GEO under accession number GSE318447.

### Bioinformatics analyses

Transcriptomic analysis was performed using publicly available NB RNA-sequencing datasets from the Gene Expression Omnibus (GEO) and Therapeutically Applicable Research to Generate Effective Treatments (TARGET) databases to investigate gene co-expression networks and potential downstream molecular mechanisms. For the identification of modules associated with high-risk and metastatic phenotypes in MYCN-nonamplified NB, weighted gene co-expression network analysis (WGCNA) was conducted using the Limma package in R to identify biologically meaningful gene modules through the online ImageGP2 platform (43). First, the expression matrix from datasets GSE49710 and GSE85047 (MYCN-nonamplified patients) was used and the differentially expressed genes between high-risk and non-high-risk groups as well as INSS stage 4 and non-stage 4 groups were acquired using the Limma package in R. The differentially expressed genes were then submitted to further WGCNA. In brief, a gene co-expression network was constructed after data preparation and filtering. Modules (pathways) were identified through hierarchical clustering and dynamic tree cut, and their expression patterns were summarized by calculating module eigengenes (MEs, the first principal component of each module). MEs were related to external phenotypic or clinical traits (*e*.*g*., disease metastasis status, disease risk groups) to identify biologically relevant modules. Finally, key drivers in the significant modules were identified for further study. The 20 cases of patients with the highest CDC20 expression and the 20 cases with the lowest CDC20 expression were selected for downstream differential gene expression (log2 FC ≥ 2, *p* < 0.05) analysis using the Limma package in R (44). Gene set enrichment analysis (GSEA) was employed to identify significantly enriched biological pathways. Genes were ranked based on their differential expression correlation. The enrichment score (ES) for each predefined gene set from the MSigDB database was calculated, reflecting the overrepresentation of its members at the ranked list's extremes. Statistical significance was determined using phenotype-based permutation testing (1000 permutations), yielding a normalized enrichment score (NES) and a false discovery rate (FDR). Gene sets with |NES| > 1.5 and FDR q-value < 0.25 were considered significantly enriched.

### Statistical analysis

All reported experimental data from three independent experiments that obey a normal distribution are shown as mean ± SD, otherwise they are described by median ± quartiles. Statistical analyses were performed using SPSS software (version 21.0; International Business Machines Corporation) or GraphPad Prism 9 software (GraphPad Software, Inc.). Normality was assessed using the Shapiro–Wilk test. Parametric or nonparametric statistical tests were selected according to whether the data satisfied the normality assumption. Two-tailed unpaired Student's *t* test was utilized to compare the means of two distinct groups of continuous variables. For data following a normal distribution, comparisons among multiple groups were performed using one-way ANOVA followed by Dunnett's or Tukey's test for *post hoc* pairwise comparisons. For data that were not normally distributed, nonparametric tests were applied: the Mann-Whitney U test for comparisons between two groups, and the Kruskal–Wallis test with Dunn's *post hoc* analysis for comparisons across more than two groups. Two-way ANOVA with appropriate multiple-comparisons tests (Dunnett or Bonferroni) was applied for experiments involving two variables or repeated conditions. Correlations were assessed using Pearson correlation. Kaplan-Meier curves and log-rank tests were used for all survival analyses. Pearson's Chi-square (*χ*2) test was applied to analyze the correlation between clinicopathological parameters and gene expression. Specific statistical tests are detailed in the corresponding figure legends.

## Ethics approval and consent to participate

All patients gave written informed consent on the use of clinical specimens for medical research. The samples used in this study were approved by the Committees for Ethical Review of Research Involving Human Subjects at the Sun Yat-Sen University Cancer Center.

## Data availability

The data supporting the findings of this study are available from the corresponding author upon reasonable request. The RNA-seq data of SK-N-SH neuroblastoma cells following CDC20 knockdown are available through the Gene Expression Omnibus (accession number GSE318447).

## Supporting information

This article contains supporting information.

## Conflict of interest

The authors declare that they have no conflicts of interest with the contents of this article.
