# Supplementary material for: GATA3-driven CDC20 activation facilitates tumor growth and metastasis through suppressing Bax/Bcl-2 ratio and boosting EMT process in neuroblastoma
Source: J Biol Chem. 2026 Sep 12;302(11):113550. doi: 10.1016/j.jbc.2026.113550 (PMC13678015; doi:10.1016/j.jbc.2026.113550)
Supplement: Supplementary file 1 [file mmc1.docx]

**Supplementary Figures and Legends**

**GATA3-driven CDC20 activation facilitates tumor growth and metastasis through suppressing Bax/Bcl-2 ratio and boosting EMT process in neuroblastoma**

Xiaona Fang^1,2^*, Yingxia Lan^2,3^*, Mengzhen Li^1,2^*, Weiji Xie^1,2^*, Lei Mao^1,2^, Jinlin Huang^2,4^, Qiuting Li^1,2^, Yu Zhang^1,2^, Mengjia Song^1,2^, Yi Que^1,2^, Suying Lu^1,2^, Juan Wang^1,2^, Junting Huang^1,2^, Jia Zhu^1,2^, Yi Wang^1,2^, Feifei Sun^1,2^, Yizhuo Zhang^1,2^.

^1^Department of Pediatric Oncology, Sun Yat-sen University Cancer Center, Guangzhou, China; ^2^State Key Laboratory of Oncology in South China, Guangdong Provincial Clinical Research Center for Cancer, Collaborative Innovation Center for Cancer Medicine, Sun Yat-sen University Cancer Center, Guangzhou, China. ^3^Department of Anesthesiology, Sun Yat-sen University Cancer Center, Guangzhou, China. ^4^Department of Pathology, Sun Yat-sen University Cancer Center, Guangzhou, China.

*These authors contributed equally to this work.

Running title: GATA3-CDC20 axis drives neuroblastoma progression.

**Correspondence:** Yizhuo Zhang. E-mail: [zhangyzh@sysucc.org.cn](mailto:zhangyzh@sysucc.org.cn)

**Figure S1. NB sample clustering and module generation using WGCNA.**

**(A)** Clustering diagram after removal of completely deviated outlier NB samples from the GSE49710 dataset. **(B)** Modules partitioned for DEGs between the high-risk and non-high-risk groups lacking MYCN amplification (GSE49710), with gray modules considered invalid. **(C)** Module partitioning using the optimal soft threshold of 9 recommended by the function. **(D)** Clustering diagram after removal of completely deviated outlier NB samples from the GSE85047 dataset. **(E)** Modules partitioned for DEGs between the metastatic (stage 4) and non-metastatic (non-stage 4) groups lacking MYCN amplification (GSE85047), with gray modules considered invalid. **(F)** Module partitioning using the optimal soft threshold of 22 recommended by the function.

**Figure S2. CDC20 is identified as an oncogene in NB and its upregulation predicts poor prognosis of patients with NB.**

**(A, B, C, D)** The expression of CDC20 was analyzed in TARGET and GEO cohorts divided according to different clinical INSS stages **(A)**, or combined MYCN status and clinical INSS stage **(B**), or stratified by COG risk group **(C)**, or MYCN status **(D).** ‘High-risk’ NB, as defined by the Children’s Oncology Group (COG) risk stratification system, includes patients with MYCN-amplified tumors, those ≥18 months of age with International Neuroblastoma Staging System (INSS) stage 4 disease, or other unfavorable clinical/biological features. Metastatic disease was defined as INSS stage 4. **(E, F, G, H)** Kaplan-Meier analysis of the OS, DFS or PFS of patients with MYCN-amplified NB **(E, F)** or high-risk NB **(G, H)** from two independent cohorts stratified by the expression level of CDC20. For the TARGET-NB cohort **(E, G)**, the best-performing threshold was selected as the cutoff value. For the SYSUCC cohort **(F, H)**, the cutoff value for stratifying NB into ‘low’ or ‘high’ CDC20 expression subgroups was calculated according to the overall survival status of patients. The P values of the log-rank tests were presented. Data were shown as mean ± SD. Statistical analyses were performed using Mann‑Whitney test for (A) and (D, Third and fourth panel), Kruskal-Wallis test with Dunn’s multiple comparisons test for (B, Left panel), one-way ANOVA with Tukey’s multiple comparisons test for (B, Right panel), two-tailed unpaired Student’s t-test for (C) and (D, First panel), two-tailed unpaired t test with Welch’s correction for (D, Second panel). Exact *p* values are indicated in the figures. A *p* value < 0.05 was considered statistically significant.

**Figure S3. Detection of CDC20 expression and overexpression of CDC20 promotes the foci formation and migration capabilities in NB cells.**

**(A, B)** Expression of CDC20 in NB cell lines and cord blood mononuclear cells (CBMCs) was detected by qRT-PCR **(A)** and Western blot analysis **(B)**. **(C)** Immunofluorescence staining shows the expression and localization of CDC20 in two NB cell lines. **(D, E)** qRT-PCR assay was used to detect *CDC20* expression in CDC20-silenced NB cells **(D)** and CDC20-overexpressing NB cells **(E). (F)** Western blot analysis was used to confirm CDC20 overexpression in NB cells. GAPDH was used as a loading control. **(G)** Representative images of colony formation in monolayer culture. The numbers of colonies were calculated and depicted in the bar chart at the right panel. **(H)** Representative images of migration (left) and quantification of migrated cells (right) from SK-N-BE(2) and SK-N-SH cells transfected with empty vector or CDC20 overexpression. Scale bars, 200 μm. Data are presented as mean ± SD from three independent biological experiments, with individual data points shown. Statistical analyses were performed using one-way ANOVA with Dunnett’s multiple comparisons test for (A) and (D), two-tailed unpaired Student’s t-test for (E), (G) and (H). Exact *p* values are indicated in the figures. A *p* value < 0.05 was considered statistically significant.

**Figure S4. CDC20 expression in normal tissues and evaluation of CDC20 dependency of Apcin activity.**

**(A)** CDC20 expression profile across normal human organs and cell types from the Genotype-Tissue Expression (GTEx) database. **(B)** Western blot analysis of Cyclin B1 and securin expression in SK-N-BE(2) and SK-N-AS cells treated with the indicated concentrations of Apcin. GAPDH was used as a loading control. **(C)** Representative images of foci formation in monolayer culture from SK-N-BE(2) cells transfected with shNC or shCDC20 in the presence of DMSO or Apcin. The numbers of foci were calculated and depicted in the bar chart at the right panel. **(D)** Representative images of migration (left) and quantification of migrated cells (right) from SK-N-BE(2) cells transfected with shNC or shCDC20 in the presence of DMSO or Apcin. Scale bars, 200 μm. **(E)** Representative flow cytometry plots and quantification of apoptosis in SK-N-BE(2) and SK-N-SH cells transduced with control vector (VEC) or CDC20 and treated with DMSO or Apcin. Apoptotic cells were detected by Alexa Fluor 647/7-AAD staining. **(F)** Representative images and quantification of transwell migration assays in SK-N-BE(2) and SK-N-SH cells transduced with control vector or CDC20 and treated with DMSO or Apcin. Scale bars, 200 μm. Data are presented as mean ± SD from three independent biological experiments, with individual data points shown. Statistical analyses were performed using one-way ANOVA with Dunnett’s multiple comparisons test and two-way ANOVA with Bonferroni’s multiple-comparisons test for (C) and (D), one-way ANOVA with Tukey’s multiple comparisons test for (E) and (F). Exact *p* values are indicated in the figures. A *p* value < 0.05 was considered statistically significant.

**Figure S5. Apcin-A suppresses proliferation and migration and induces apoptosis in NB cells.**

**(A)** Dose–response analysis of Apcin-A in SK-N-BE(2) and SK-N-SH cells. Cell viability was measured following treatment with the indicated concentrations of Apcin-A, and IC50 values were calculated from nonlinear dose–response curves. **(B)** Time-course analysis of NB cell proliferation following treatment with DMSO or Apcin-A. **(C)** Representative flow cytometry plots and quantification of apoptosis in SK-N-BE(2) and SK-N-SH cells treated with DMSO or Apcin-A. Apoptotic cells were detected by Alexa Fluor 647/7-AAD staining. **(D)** Representative images and quantification of transwell migration assays in SK-N-BE(2) and SK-N-SH cells treated with DMSO or Apcin-A at the indicated concentrations. Scale bars, 200 μm. Data are presented as mean ± SD from three independent biological experiments, with individual data points shown. Statistical analyses were performed using two-way ANOVA with Bonferroni’s multiple-comparisons test for (B) and two-tailed unpaired Student’s t-test for (C-D). Exact *p* values are indicated in the figures. A *p* value < 0.05 was considered statistically significant.

**Figure S6. The expression of GATA3 in normal cell line, NB cell lines and NB tissues.**

**(A, B)** Expression of GATA3 in NB cell lines and cord blood mononuclear cells (CBMCs) was detected by qRT-PCR **(A)** or Western blot analysis **(B)**. **(C)** Immunofluorescence staining shows the expression and localization of GATA3 in SK-N-BE(2) and SK-N-SH cells. **(D)** Representative images from IHC staining showing the distinct levels of GATA3 protein expression in NB clinical specimens. Scale bars, 50 μm. **(E)** The expression of GATA3 was analyzed in the GEO cohort (GSE66586), including normal neuronal cells (HCN1-A) and human bone marrow-derived mesenchymal stem cells (hBM-MSCs), as well as various MYCN-amplified and -nonamplified NB cell lines. **(F, G)** The expression of GATA3 was analyzed in TARGET and GEO cohorts stratified according to MYCN status **(F)** or combined MYCN status and COG risk group **(G)**. ‘High-risk’ NB, as defined by the Children's Oncology Group (COG) risk stratification system, includes patients with MYCN-amplified tumors, those ≥18 months of age with International Neuroblastoma Staging System (INSS) stage 4 disease, or other unfavorable clinical/biological features. Data were shown as mean ± SD. Statistical analyses were performed using one-way ANOVA with Dunnett’s multiple comparisons test for (A), one-way ANOVA with Tukey’s multiple comparisons test for (E), Mann‑Whitney test for (F), and Kruskal-Wallis test with Dunn’s multiple comparisons test for (G). Exact *p* values are indicated in the figures. A *p* value < 0.05 was considered statistically significant.

**Figure S7. Knockdown of GATA3 impairs the cell proliferation and migration capabilities and induces apoptosis in NB cells.**

**(A)** Western blot analysis was used to detect GATA3 expression in GATA3-silenced NB cells. β-actin was used as a loading control. **(B)** The CCK-8 assay was utilized to determine the NB cell proliferation rates. **(C)** Representative images of foci formation in monolayer culture (left). The numbers of foci were calculated and depicted in the bar chart (right). **(D)** Representative flow cytometry plots (left) and summary (right) of apoptotic cells in SK-N-BE(2) and SK-N-SH cells transfected with siNC or siGATA3. **(E)** Representative images of wound healing assay in SK-N-BE(2) and SK-N-SH cells transfected with siNC or siGATA3. Scale bars, 200 μm. **(F)** The migration rate of cells was illustrated in the bar chart. Data are presented as mean ± SD from three independent biological experiments, with individual data points shown. Statistical analyses were performed using two-way ANOVA with Dunnett’s multiple comparisons test for (B) and one-way ANOVA with Dunnett’s multiple comparisons test for (C), (D) and (F). Exact *p* values are indicated in the figures. A *p* value < 0.05 was considered statistically significant.

**Figure S8. Knockdown or overexpression of CDC20 has no effect on the expression of GATA3 in NB cells.**

**(A)** qRT-PCR assay showing the effect of CDC20 knockdown on the mRNA levels of *GATA3* in SK-N-BE(2) and SK-N-SH cells. **(B)** Western blot analysis showing the effect of CDC20 knockdown on the protein levels of GATA3 in SK-N-BE(2) and SK-N-SH cells. **(C)** qRT-PCR analysis showing the effect of CDC20 overexpression on the mRNA levels of *GATA3* in SK-N-BE(2) and SK-N-SH cells. Data are presented as mean ± SD from three independent biological experiments, with individual data points shown. Statistical analyses were performed using one-way ANOVA with Dunnett’s multiple comparisons test for (A) and two-tailed unpaired Student’s t-test for (C). Exact *p* values are indicated in the figures. A *p* value < 0.05 was considered statistically significant.

**Figure S9. The GATA3–CDC20 axis is associated with EMT-like changes and apoptotic resistance in NB cells.**

**(A)** The KEGG enrichment analysis based on 220 upregulated genes in CDC20^high^ NB tissues in contrast to CDC20^low^ NB tissues. **(B)** The hallmark enrichment analysis based on 342 downregulated genes upon CDC20 knockdown in SK-N-SH cells. **(C)** The GO-BP enrichment analysis based on 342 down-regulated genes upon CDC20 knockdown in SK-N-SH cells. **(D)** qRT-PCR assay showing the effect of CDC20 knockdown on the mRNA levels of multiple epithelial and mesenchymal markers in SK-N-BE(2) and SK-N-SH cells. **(E)** Heat-map depicts the effect of CDC20 knockdown on the mRNA expression of ADRN- and MES-related transcription factors in SK-N-SH cells. **(F)** qRT-PCR assay showing the effect of CDC20 knockdown on the mRNA levels of multiple apoptosis-related genes in SK-N-SH cells. **(G)** Western blot analysis showing the effect of GATA3 knockdown on the protein levels of multiple epithelial and mesenchymal markers in SK-N-BE(2) and SK-N-AS cells. **(H)** qRT-PCR assay showing the effect of GATA3 knockdown on the mRNA levels of multiple epithelial and mesenchymal markers in SK-N-BE(2) and SK-N-AS cells. Data are presented as mean ± SD from three independent biological experiments, with individual data points shown. Statistical analyses were performed using one-way ANOVA with Dunnett’s multiple comparisons test for (D) and (H), two-tailed unpaired Student’s t-test for (F). **p*< 0.05, ***p*< 0.01, ****p*< 0.001, *****p*< 0.0001; ns, not significant.
