# Supplementary material for: GATA3-driven CDC20 activation facilitates tumor growth and metastasis through suppressing Bax/Bcl-2 ratio and boosting EMT process in neuroblastoma
Source: J Biol Chem. 2026 Sep 12;302(11):113550. doi: 10.1016/j.jbc.2026.113550 (PMC13678015; doi:10.1016/j.jbc.2026.113550)
Supplement: Supplementary file 2 [file mmc2.docx]

**Table S1. The list of 46 overlapping candidates.**

**Candidate genes**

DLGAP5

KIF18A

BRIP1

CDCA2

KIF14

CDC45

KIF4A

ARHGAP11A

ANLN

DTL

FANCI

CENPF

CCNA2

EXO1

CCNB1

TOP2A

KIF2C

BUB1

FOXM1

ASPM

NCAPG

AURKA

GTSE1

CEP55

FAM111B

ATAD2

CDC6

TICRR

BLM

KIF20A

HJURP

CDCA5

CDCA8

CLSPN

E2F8

BRCA2

BIRC5

FAM83D

GINS2

CDKN3

CDC20

DSCC1

CENPE

CHAF1A

CENPA

E2F1
