# Supplementary material for: GATA3-driven CDC20 activation facilitates tumor growth and metastasis through suppressing Bax/Bcl-2 ratio and boosting EMT process in neuroblastoma
Source: J Biol Chem. 2026 Sep 12;302(11):113550. doi: 10.1016/j.jbc.2026.113550 (PMC13678015; doi:10.1016/j.jbc.2026.113550)
Supplement: Supplementary file 3 [file mmc3.docx]

**Table S2. Predicted binding sequences of CDC20 promoter regions by transcription factor GATA3 via JASPAR database.**

| **Matrix ID** | **Name** | **Score** | **Relative score** | **Sequence ID** | **Start** | **End** | **Strand** | **Predicted sequence** |
| --- | --- | --- | --- | --- | --- | --- | --- | --- |
| [MA0037.3](https://jaspar.elixir.no/matrix/MA0037.3) | MA0037.3.GATA3 | 10.987059 | 0.9738492 | NC_000001.11:43356981-43358981 | 1262 | 1269 | - | AGATAAGC |
| [MA0037.3](https://jaspar.elixir.no/matrix/MA0037.3) | MA0037.3.GATA3 | 10.074881 | 0.95287323 | NC_000001.11:43356981-43358981 | 352 | 359 | + | AGATAATA |
| [MA0037.3](https://jaspar.elixir.no/matrix/MA0037.3) | MA0037.3.GATA3 | 9.685181 | 0.94391185 | NC_000001.11:43356981-43358981 | 361 | 368 | - | AGATAATG |
| [MA0037.3](https://jaspar.elixir.no/matrix/MA0037.3) | MA0037.3.GATA3 | 8.782075 | 0.9231445 | NC_000001.11:43356981-43358981 | 527 | 534 | - | AGATTAGA |
| [MA0037.3](https://jaspar.elixir.no/matrix/MA0037.3) | MA0037.3.GATA3 | 8.56087 | 0.91805786 | NC_000001.11:43356981-43358981 | 609 | 616 | + | CGATAATG |
| [MA0037.3](https://jaspar.elixir.no/matrix/MA0037.3) | MA0037.3.GATA3 | 7.61301 | 0.8962614 | NC_000001.11:43356981-43358981 | 1273 | 1280 | + | AGATAGGC |
