# Supplementary material for: GATA3-driven CDC20 activation facilitates tumor growth and metastasis through suppressing Bax/Bcl-2 ratio and boosting EMT process in neuroblastoma
Source: J Biol Chem. 2026 Sep 12;302(11):113550. doi: 10.1016/j.jbc.2026.113550 (PMC13678015; doi:10.1016/j.jbc.2026.113550)
Supplement: Supplementary file 4 [file mmc4.docx]

**Table S3. Oligonucleotides used for plasmid construction and transient transfection.**

| **Gene** | **Oligo name** | **Strand** | **Oligo Sequence (5‘-3’)** |
| --- | --- | --- | --- |
| CDC20 | siRNA-1 | Sense | GGAAGACCUGCCGUUACAUTT |
|  |  | Antisense | AUGUAACGGCAGGUCUUCCTT |
|  | siRNA-3 | Sense | GCACAGAACCAGCUAGUUATT |
|  |  | Antisense | UAACUAGCUGGUUCUGUGCTT |
| GATA3 | siRNA-1 | Sense | UCUGGAGGAGGAAUGCCAATT |
|  |  | Antisense | UUGGCAUUCCUCCUCCAGATT |
|  | siRNA-2 | Sense | CCCUGACUAUGAAGAAGGATT |
|  |  | Antisense | UCCUUCUUCAUAGUCAGGGTT |
| CDC20 | shRNA1 | / | CCGGGCAGAAACGGCTTCGAAATATCTCGAGATATTTCGAAGCCGTTTCTGCTTTTTT |
|  | shRNA3 | / | CCGGCTAAGCTGGAACAGCTATATCCTCGAGGATATAGCTGTTCCAGCTTAGTTTTTT |
| GATA3 | shRNA1 | / | CCAAGAACAGCTCGTTTAACC |
|  | shRNA2 | / | GCCGTCTTGATACTTTCAGAA |
|  | shRNA3 | / | GGGAGAAATAGTGTGGAAATT |
