# Supplementary material for: GATA3-driven CDC20 activation facilitates tumor growth and metastasis through suppressing Bax/Bcl-2 ratio and boosting EMT process in neuroblastoma
Source: J Biol Chem. 2026 Sep 12;302(11):113550. doi: 10.1016/j.jbc.2026.113550 (PMC13678015; doi:10.1016/j.jbc.2026.113550)
Supplement: Supplementary file 5 [file mmc5.docx]

**Table S4. Primers used for qRT-PCR assays.**

| **Gene** | **Strand** | **Sequence (5’-3’)** |
| --- | --- | --- |
| ACTB | Forward | CCTGGCACCCAGCACAAT |
|  | Reverse | GGGCCGGACTCGTCATAC |
| GAPDH | Forward | GGACCTGACCTGCCGTCTAG |
|  | Reverse | GTAGCCCAGGATGCCCTTGA |
| CDC20 | Forward | AAGACCTGCCGTTACATTCCTTCC |
|  | Reverse | CACCAGAGCTTGCACTCCACAG |
| GATA3 | Forward | CACCACCTACCCGCCCTACG |
|  | Reverse | GTTCACACACTCCCTGCCTTCTG |
| SNAI1 | Forward | ACATCCGAAGCCACACG |
|  | Reverse | TGGGGACAGGAGAAGGG |
| SNAI2 | Forward | CTGCGATGCCCAGTCTA |
|  | Reverse | CCCCGTGTGAGTTCTAATG |
| VIM | Forward | GACGCCATCAACACCGAGTT |
|  | Reverse | CTTTGTCGTTGGTTAGCTGGT |
| ZEB1 | Forward | GACCACAGATACGGCAAAA |
|  | Reverse | CTGAAGCAACCACTATGGG |
| CDH2 | Forward | TGCGGTACAGTGTAACTGGG |
|  | Reverse | GAAACCGGGCTATCTGCTCG |
| CDH1 | Forward | CGAGAGCTACACGTTCACGG |
|  | Reverse | GGGTGTCGAGGGAAAAATAGG |
| CD44 | Forward | TCAGACCCACTCAGCCA |
|  | Reverse | GCTCCTCAGCTTCCAACTC |
| PROM1 | Forward | GCATCCATCAAGTGAAACC |
|  | Reverse | ACCAGGCCATCCAAATC |
| CCNA1 | Forward | GGCCAGGGGATCACAAGAAT |
|  | Reverse | CTTGAGTGTGCCGGTGTCTA |
| CASP3 | Forward | AGATGGTTTGAGCCTGAGCA |
|  | Reverse | GTGCGTATGGAGAAATGGGC |
| CASP8 | Forward | TGGGAATGTTTTTAGCTGGTGG |
|  | Reverse | GTGGTAGTGTGGAATTTAGCTTTGA |
| CASP10 | Forward | CAGTGGCTTGGGTTCACAGT |
|  | Reverse | ACAGAACACGAAGCAGTCCC |
| ERBB3 | Forward | TGAATGGCCTGAGTGTGACC |
|  | Reverse | AAGGAGAGGTCGGCATTGTG |
| LGALS3 | Forward | GGCCTACCCATCTTCTGGAC |
|  | Reverse | CCCAGGCAAAGGCAGGTTAT |
| BCL2L10 | Forward | CGGTCCTTTTTCTCCGCCTA |
|  | Reverse | TCACGAGCGTCACCACTCT |
| BCL2 | Forward | TCTTTGAGTTCGGTGGGGTC |
|  | Reverse | TCCACAGGGCGATGTTGTC |
| BAX | Forward | TTCTGACGGCAACTTCAACT |
|  | Reverse | CAGCCCATGATGGTTCTGAT |
