# Supplementary material for: GATA3-driven CDC20 activation facilitates tumor growth and metastasis through suppressing Bax/Bcl-2 ratio and boosting EMT process in neuroblastoma
Source: J Biol Chem. 2026 Sep 12;302(11):113550. doi: 10.1016/j.jbc.2026.113550 (PMC13678015; doi:10.1016/j.jbc.2026.113550)
Supplement: Supplementary file 6 [file mmc6.docx]

**Table S5. Primers used for ChIP-qPCR assays.**

| Gene | Strand | Sequence (5’-3’) |
| --- | --- | --- |
| CDC20 Promoter 1 | Forward | TTCCCCACCTCTAACCTAAGCAT |
|  | Reverse | CCAGAAAGAGGCTGGAAAAATCA |
| CDC20 Promoter 2 | Forward | ATCTATCTGCCTTATGGGATT |
|  | Reverse | GAGCGATACTCTGTCTGAAAA |
| CDC20 Promoter 3 | Forward | TCCCAAGCTTCCCAATTCCG |
|  | Reverse | GTGCAGAACCCGATGGAGTG |
| CDC20 Promoter 4 | Forward | TACCCTTCAAATCGCGCTCC |
|  | Reverse | AAAATCCAACCGTCGCAACC |
